# Investigating the therapeutic potential of hesperidin targeting CRISP2 in intervertebral disc degeneration and cancer risk mitigation

**DOI:** 10.3389/fphar.2024.1447152

**Published:** 2024-08-29

**Authors:** Hui Zhang, Wei Jiang, Yuqing Jiang, Nanwei Xu, Luming Nong, Tengfei Li, Ruiping Liu

**Affiliations:** ^1^ Department of Orthopedics, The Affiliated Changzhou Second People’s Hospital of Nanjing Medical University, Changzhou, Jiangsu, China; ^2^ Changzhou Medical Center, Nanjing Medical University, Changzhou, Jiangsu, China; ^3^ Department of Orthopedics, Gonghe County Hospital of Traditional Chinese Medicine, Hainan, Qinghai, China; ^4^ Graduate School, Tianjin Medical University, Tianjin, China

**Keywords:** hesperidin, intervertebral disc degeneration, CRISP2, estrogen therapy, inflammation, natural metabolic treatment

## Abstract

**Background:**

Intervertebral disc degeneration (IDD) can lead to disc herniation and spinal instability, sometimes requiring surgical intervention. Currently, estrogen has a potential protective effect on IDD, and estrogen is associated with an increased risk of some cancers, such as breast and endometrial cancer. Therefore, it is important to identify natural compounds that estrogen analogues treat IDD while reducing the risk of tumor development.

**Objective:**

This study aims to explore a natural metabolic treatment strategy by targeting CRISP2 with the natural compound Hesperidin to mimic the protective effects of estrogen on IDD and reduce the risk of tumor development.

**Methods:**

Microarray data from healthy volunteers and IDD patients were extracted from the Gene Expression Omnibus (GEO) database, and RNA sequencing and clinical data from various cancer types were analyzed. Differentially expressed genes (DEGs) were identified using the Bioconductor Limma package, followed by principal component analysis, volcano plot, and heatmap visualization. Additionally, Gene Ontology (GO) and Kyoto Encyclopedia of Genes and Genomes (KEGG) analyses, CIBERSORT and ssGSEA immune cell infiltration assessments, survival analysis, metabolite enrichment analysis, and molecular docking were performed. Hesperidin’s interaction with CRISP2 was further validated through molecular docking and experimental studies.

**Results:**

Hesperidin significantly reduced the expression of CRISP2, iNOS, and COX2 in IDD models, decreased reactive oxygen species (ROS) and apoptosis, and diminished inflammatory markers. CIBERSORT and ssGSEA analyses revealed a correlation between CRISP2 and immune cell infiltration. Survival analysis demonstrated that CRISP2 expression levels were associated with patient survival across various cancer types. Hesperidin was found to mimic estrogen’s effects on IDD and reduce tumor progression. Cell culture and experimental validation confirmed Hesperidin’s protective effects on nucleus pulposus cells (NPCs).

**Conclusion:**

Hesperidin, as a potential natural metabolic regulator, not only has therapeutic effects on IDD but may also synergize with estrogen therapy to promote spinal health without increasing cancer risk. This study presents a new clinical approach for IDD treatment and lays the foundation for further drug development and experimental research.

## Graphical Abstract

**Figure:**
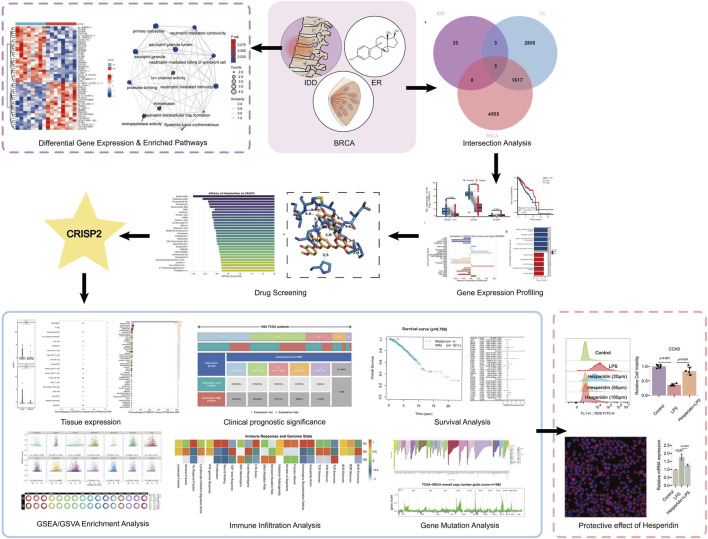
The graphic abstract illustrates a study investigating the therapeutic potential of Hesperidin in targeting the CRISP2 gene to treat intervertebral disc degeneration (IDD) while mitigating cancer risks associated with estrogen. The workflow begins with differential gene expression and enriched pathway analysis in IDD, breast cancer (BRCA), and estrogen receptor (ER) pathways. An intersection analysis reveals overlapping genes, highlighting CRISP2 as a significant target. Hesperidin emerges as a promising natural metabolic regulator for treating IDD and potentially collaborating with estrogen therapy to improve spinal health while minimizing cancer risks, paving the way for innovative therapeutic approaches.

## Highlights

• Discovery of Hesperidin as a natural metabolic regulator targeting CRISP2 to mimic estrogen’s protective effects on Intervertebral Disc Degeneration (IDD) while reducing tumor progression risk.
• Comprehensive bioinformatics analysis reveals Hesperidin’s anti-inflammatory and antioxidative properties, reducing CRISP2, iNOS, COX2 expression, and reactive oxygen species (ROS) in IDD models.
• Experimental validation confirms Hesperidin’s protective effects on nucleus pulposus cells (NPCs), highlighting its potential as a safer alternative to estrogen therapy for spinal health.

## Background

Intervertebral Disc Degeneration (IDD) is a significant pathological condition characterized by the deterioration of disc structure and function, often resulting in lower back pain and nerve root compression (Ravalli and Musumeci, 2021; Ravalli and Musumeci, 2022). Globally, about 80% of people experience lower back pain during their lifetime, with IDD being a major contributor (Schochat and Jäckel, 1998; Hoy et al., 2010). Despite its prevalence, the etiology and pathomechanisms of IDD remain incompletely understood, underscoring the need for in-depth resreach to advance clinical treatments (Oichi et al., 2020; Iwase et al., 2017). The degenerative process of IDD includes the destruction of the extracellular matrix, loss of disc height, and inflammation, often leading to disc herniation and spinal instability, sometimes necessitating surgical intervention (Yao et al., 2023; Kos et al., 2019).

Current treatment options for IDD are primarily symptomatic and lack efficacy in halting or reversing the degenerative process (Krut et al., 2021). Thus, there is a pressing need for novel therapeutic approaches that address the underlying molecular mechanisms of IDD (Kamali et al., 2021; Fuertes et al., 2019). Understanding the mechanisms of inflammation and its regulation could provide insights into potential therapeutic strategies for IDD (Liu Z. et al., 2023; Morsi et al., 2015). The systemic immune inflammation index has been shown to predict adverse cardiovascular outcomes, highlighting the critical role of inflammation in disease progression (Yang et al., 2020; Moriya, 2019). Estrogen supplementation has been recognized for its potential protective effects against IDD (Christianson et al., 2015; Howard and Rossouw, 2013). Estrogen’s interaction with specific receptors is thought to play a crucial role in maintaining disc integrity (Song et al., 2017; Sheng et al., 2018). However, the use of estrogen is associated with increased risks of certain cancers, such as breast and endometrial cancers, presenting a significant challenge in therapeutic approaches involving estrogen. This paradox necessitates finding alternative solutions that provide the protective benefits of estrogen while mitigating the associated cancer risks (Mueck and Seeger, 2015).

A promising approach lies in identifying natural compounds that could mimic the protective effects of estrogen on IDD while reducing the risk of tumor development (Asare et al., 2017). Human metabolites, as endogenous compounds, offer a potential avenue for such interventions (Descamps et al., 2019; Gonzalez-Covarrubias et al., 2022). Several studies have investigated the relationship between estrogen, gene expression in IDD, and cancer risks (Tanaka et al., 2007). However, comprehensive research exploring human metabolites that could serve both protective and anti-tumor roles is lacking. Previous research has primarily focused on singular aspects of estrogen’s effects, either on IDD or cancer, without a holistic approach considering both outcomes.

Cancer is a complex disease involving multiple processes, including epithelial-mesenchymal transition (EMT) (Zhang and Weinberg, 2018). Insights into the mechanism and dynamic regulation of EMT in ovarian cancer (Prayudi et al., 2023) may help us understand the increased cancer risk associated with estrogen therapy. This understanding is crucial for developing further therapeutic strategies (Fan et al., 2020). Long non-coding RNAs (lncRNAs) have emerged as promising therapeutic targets and biomarkers for ischemic stroke, with potential implications for cancer therapy (Fan et al., 2020; Zhong et al., 2023).

Recent advances in disc biology have highlighted the role of metabolic dysregulation in IDD pathogenesis (Zhu et al., 2024; Che et al., 2022). Metabolic regulators can restore the balance between anabolic and catabolic activities within disc cells, potentially serving as therapeutic agents (Byrnes et al., 2022). Natural compounds are particularly noteworthy for their safety and multi-target effects. Recent studies have emphasized the potential of natural compounds in treating various diseases. However, specific metabolic regulators that effectively combat disc degeneration remain to be fully elucidated (Diederich, 2020).

Emerging research suggests that certain natural metabolic modulators can mimic estrogen’s effects, offering dual benefits in treating IDD and reducing cancer risk (Jordan and O’Malley, 2007). Nevertheless, the specific mechanisms by which these modulators impact disc health and tumor progression are not fully understood (Heggli et al., 2024; Borza and Pozzi, 2014). Additionally, there is a lack of comprehensive bioinformatics analysis in the current literature to identify and validate these modulators (Cox, 2015). Therefore, identifying natural compounds that can mimic estrogen’s therapeutic effects on IDD while minimizing its oncogenic potential is of significant clinical importance.

Bioinformatics and multi-omics approaches play an increasingly vital role in disease treatment research, providing tools to uncover genetic and epigenetic modifications involved in disease progression (Dar et al., 2023; Lu et al., 2015; Sun and Hu, 2016). Through big data analysis and biomarker identification, researchers can gain deeper insights into disease mechanisms and innovate new therapeutic approaches (Liu, 2024; Berger et al., 2023). Studies on predictive factors for empty follicle syndrome in infertile patients undergoing assisted reproductive technology highlight the relevance of bioinformatics in disease risk prediction (Ali et al., 2022). The genome-wide identification and expression analysis of ARF and AUX/IAA gene families in soybeans offer insights into gene expression regulation, relevant to gene expression analysis and multi-omics studies (Ali et al., 2022; Liu et al., 2021). Furthermore, transcriptomic analyses are crucial for understanding the immune microenvironment’s role in the diagnosis and prognosis of various diseases (Martio et al., 2023; Zhou et al., 2023; Mei et al., 2024). Additionally, transcriptomic evaluation in uncovering the role of immune microenvironment is vital for diagnosing and prognosticating multiple diseases (Martio et al., 2023; Wu et al., 2023; Huang et al., 2013). Recent research has demonstrated that through precise biomolecular regulation and the application pf specific compounds, significant biological responses can be achieved, providing potential therapeutic targets and strategies (Du and Liu, 2024; Yao et al., 2021; Biasutto et al., 2019). Traditional drug formulations have also shown promise in improving diagnostics and health management through extensive data analysis and bioinformatics (Yao et al., 2024; Qian et al., 2019). Bioinformatics plays a crucial role in analyzing gene expression and regulatory mechanisms, providing essential insights into biological processes (Du and Liu, 2024; Yan et al., 2022; Jin and Qu, 2017). In conclusion, this research not only deepens the understanding of molecular mechanisms but also provides a crucial foundation for developing novel therapeutic strategies (Liang et al., 2023; Wan et al., 2024; Liu P. et al., 2023; Chen et al., 2022).

The development of novel targeted therapeutic strategies is expected to enhance treatment effectiveness and minimize side effects, thereby advancing precision medicine (Wang and Wang, 2024; Guan et al., 2024; Hu et al., 2024; Watanabe and Seki, 2024). The current study aims to address these gaps by using bioinformatics techniques to identify natural metabolic modulators that can mimic estrogen’s therapeutic effects on IDD while reducing tumor progression (Liao et al., 2022). Specifically, we will analyze gene expression profiles and metabolic pathways involved in IDD, estrogen therapy, and cancer to identify candidate compounds (Deng et al., 2022; Boehme et al., 2009). Subsequent experimental validation will assess the therapeutic potential and safety of these compounds (Blass, 2015). We have identified the compound hesperidin and key regulatory factor Cysteine-Rich Secretory Protein 2 (CRISP2). Hesperidin is a bioflavonoid found predominantly in citrus fruits, known for its antioxidant and anti-inflammatory properties. Recent studies have suggested its potential in modulating various pathological conditions, including those involving oxidative stress and inflammation. In the context of IDD, Hesperidin’s ability to reduce oxidative damage and inflammatory responses could provide a therapeutic advantage. CRISP2 is involved in cellular processes such as proliferation, apoptosis, and extracellular matrix maintenance. Emerging evidence suggests that CRISP2 plays a role in maintaining disc cell integrity. Dysregulation of CRISP2 expression has been implicated in disc degeneration, highlighting its potential as a biomarker and therapeutic target for IDD.

This study aims to explore the effects of Hesperidin on IDD by examining its impact on oxidative stress and inflammation, and its interaction with CRISP2. By elucidating these mechanisms, we seek to establish a basis for the therapeutic potential of Hesperidin in managing IDD and improving patient outcomes. By achieving these objectives, this study aims to deepen the understanding of IDD treatment and propose new therapeutic strategies that maximize efficacy while minimizing adverse effects. The study’s findings can inform clinical practice and policy-making, promoting the development of more effective and safer IDD treatment modalities.

## Materials and methods

### Data acquisition and differential analysis

For this study, microarray data were extracted from the Gene Expression Omnibus (GEO) database (GSE124272), comprising samples from eight healthy volunteers and eight patients with IDD. Additionally, RNA sequencing and clinical data from 33 different cancer types were analyzed, sourced from the BRCA-TCGA database. Using the Bioconductor Limma package, we identified differentially expressed genes (DEGs) from GSE124272, applying a selection criterion of p < 0.05 and |log2 (fold change) | > 1.5. Principal component analysis (PCA) was performed to explore the variations among the samples, and the heatmap package was used to generate volcano and heat maps for visualizing the DEGs.

### Functional and pathway correlation analysis

To comprehensively analyze the functions of the DEGs, we used the clusterProfiler package for Gene Ontology (GO) analysis and Kyoto Encyclopedia of Genes and Genomes (KEGG) pathway analysis. In the GO analysis, both the p-value and q-value thresholds were set to less than 0.05. For the KEGG pathway analysis, results were considered significantly enriched if the q-value was below 1.00.

### CIBERSORT and ssGSEA immune cell infiltration assessment

We employed the CIBERSORT deconvolution algorithm to estimate the abundance of 22 unique immune cell types, assessing the proportion of various immune cells across the 16 samples in the GSE124272 dataset. Differences in immune cell proportions between IDD and control samples were also evaluated. Visualization was accomplished using the “corrplot” and “vioplot” packages to create stacked bar charts, with an adjusted P-value threshold of less than 0.05. Additionally, we calculated the Pearson correlation coefficients between CRISP2 and various immune cell types using the CIBERSORT method to elucidate the strength and direction of these relationships.

Furthermore, we applied single-sample Gene Set Enrichment Analysis (ssGSEA) to evaluate the correlation between CRISP2 expression and different immune cell types. GSEA is a computational approach used to assess the expression status of specific gene sets within a sample. We first defined gene sets specific to various immune cell types using markers from public databases such as ImPort. Then, we calculated enrichment scores for these gene sets in each sample using the ssGSEA algorithm, determining the Pearson correlation coefficients between CRISP2 expression and the enrichment scores of different immune cell types.

### Survival analysis and evaluation of diagnostic value for key genes

We utilized Kaplan-Meier survival analysis to examine the relationship between overall survival (OS) in patients across 33 different cancer types and the expression levels of key mRNA. To assess the ability of key genes to differentiate between IDD and control samples within the GSE124272 dataset, we applied the pROC R package to determine their diagnostic value.

### Metabolite enrichment analysis via MetaboAnalyst

This module accepts a list of compound names, concentration data, or a concentration table. The analysis utilizes 15 libraries containing around 13,000 biologically relevant metabolite sets, primarily sourced from human studies and including over 1,500 chemical categories. The process begins by creating a concentration-based analysis data object within the MetaboAnalyst platform. Compounds are mapped to the database using the MapData and CrossReferencing functions. The CreateMappingResultTable function is then used to compile the mapping results. The metabolome filter is set to include all detected metabolites. The pathway library is configured using the SetCurrentMsetLib function with the “ramp_path” setting. Finally, the CalculateHyperScore function calculates enrichment scores for the identified pathways.

### Molecular docking

Compound libraries are essential tools for drug screening and significantly influence the speed and quality of small molecule drug development. The metabolite data for this study were sourced from MedChemExpress LLC, and all these small molecules underwent molecular docking and virtual screening. The analysis included metabolite set enrichment analysis (MSEA) using MetaboAnalyst 6.0, incorporating human, mammalian, and chemical class metabolite sets. The structure for docking CRISP2 was obtained from the RCSB database, while the three-dimensional structures of small molecules were downloaded from the PubChem database. Molecular docking was performed using AutoDock Vina 1.1.2 software. Prior to docking, all receptor proteins were prepared using the academic open-source version of PyMol. The processed protein and small molecule PDBQT files were then input into Vina for docking. Finally, the docking results were visualized using PyMol academic open-source version, and detailed visualization was performed using PLIP, an automated protein-ligand interaction profiler (Salentin et al., 2015).

### Pan-cancer expression landscape of CRISP2

In this study, we employed the Wilcoxon rank-sum test to evaluate the significance of gene expression differences between tumor and normal tissue samples. Data were sourced from the TCGA project and standardized using the PanCanAtlas database. The specific dataset, EBPlusPlusAdjustPANCAN_IlluminaHiSeq_RNASeqV2.geneExp.tsv, was generated by the Firehose analysis pipeline utilizing MapSplice and RSEM algorithms. To ensure data comparability, raw data were normalized with the upper quartile set to 1,000, and Z-Scores were calculated to transform the data into dimensionless standardized scores for improved uniformity. The study also integrated data from the HPA and GTEx projects to create an RNA consensus tissue gene expression database, encompassing gene expression levels across 50 different tissues, quantified using nTPM values. For multi-subtissue structures like the brain, lymph, and intestines, the highest expression value among the subtissues was used. The foundational data included HPA version 23.0 and Ensembl version 109, along with protein localization information from immunofluorescence staining. The resulting dataset, in tab-delimited format, contained gene identifiers, names, reliability scores, localization data, cell cycle dependency, and GO cellular component term identifiers. Records with null values were removed to ensure accuracy. Additionally, gene expression was analyzed across 81 cell types in 31 datasets from the HPA database, with a focus on 18 cell types and PBMC expression. This analysis summarized gene expression patterns in 28 cancer types, highlighting changes in gene expression during cancer progression.

### Clinical prognostic significance of CRISP2 in BRCA

This study investigates significant gene expression differences between tumor and normal tissues using two datasets from the UCSC Xena database: “tcga_RSEM_gene_tpm,” representing TPM expression levels in TCGA tumor samples, and “gtex_RSEM_gene_tpm,” representing TPM expression levels in normal samples from the GTEx project. The data were standardized using Z-Score normalization to eliminate dimensional differences and enhance comparability. Outliers with absolute Z-Score values greater than 3.0 were excluded during preprocessing to minimize their impact on the analysis results. We employed the Wilcoxon rank-sum test, a non-parametric statistical method suitable for non-normally distributed data, to evaluate the statistical differences in gene expression between tumor and normal tissues. Additionally, the “pROC” package was used to conduct ROC analysis, assessing the diagnostic efficacy of specific gene expression levels by calculating the 95% confidence interval (CI), Area Under the Curve (AUC), and plotting the ROC curve to quantify the gene expression’s effectiveness in distinguishing between tumor and normal tissues. Tumor samples were categorized into high and low expression groups based on the median gene expression value. The distribution proportions of these groups across different molecular subtypes were analyzed using the chi-square test to determine the statistical significance of distribution differences. Furthermore, a Kruskal-Wallis rank-sum test was performed to assess variations in CRISP2 gene expression among various molecular immune subtypes within the BRCA dataset.

### CRISP2 survival analysis

In this study, we investigated the impact of gene expression levels on patient survival using Kaplan-Meier survival analysis. Detailed survival data were analyzed with the “survival” package in R, while the “survminer” package was employed to determine optimal cut-off values for high and low expression groups, ensuring that each group included at least 30% of the sample size to enhance statistical power. Additionally, we utilized the inverse variance method to perform a meta-analysis of univariate Cox proportional hazards models, synthesizing results from multiple studies. The hazard ratio (HR) was used as the primary effect measure, categorized into HR < 1 and HR > 1 groups to represent potential tumor-suppressive and tumor-promotive effects, respectively, thereby simplifying the complex relationship between gene expression and biological mechanisms. Statistical analysis and visualization were conducted using the ‘meta’ package in R version 4.3.2, which includes features for generating forest plots and funnel plots to display combined effect sizes and assess publication bias.

### Single-gene GSVA enrichment analysis for CRISP2

In this examination, we used a stratified technique to categorize samples into high and low expression groups, defining the top 30% of samples in terms of expression as the high expression group and the bottom 30% as the low expression group. This method helps in identifying significant changes in gene expression under disease conditions and exploring their biological implications. We applied the “limma” package, a widely used tool in R for differential expression analysis, to calculate the log2 fold change (log2FC) for each gene and rank them to identify significantly altered genes. Further, we implemented the z-score algorithm proposed by Lee et al. to assess the activity of biological pathways by integrating the expression of specific gene sets. Using the z-score algorithm within the “GSVA” package in R, we analyzed 14 functional state gene sets, converting their expression values into z-scores. To delve deeper into the relationship between gene expression and functional states, we performed a Pearson correlation analysis to evaluate the statistical correlation between gene expression and gene set z-scores. Additionally, we employed the “gsva” function within the GSVA package to score 73 metabolic gene sets from the KEGG database and used the “limma” package to compare the metabolic pathway activities between high and low expression groups, aiming to reveal the roles of these pathways in disease progression. For clinical variable analysis, patients were divided into high and low expression groups based on the median expression value, and age groups were determined using the median age as the threshold. The chi-square test was used to examine the distribution differences of various clinical variables between the two expression groups.

### CRISP2 immune infiltration analysis

This study utilized immune infiltration data from the TIMER 2.0 database, which compiles records from the TCGA project. TIMER 2.0 is an integrated platform employing multiple algorithms to evaluate the composition of immune cells within the tumor microenvironment and their correlation with gene expression. This approach ensured data accuracy and consistency, allowing for a comprehensive assessment of the relationship between immune cells and gene expression. We visualized the correlation coefficients between different immune cell types and gene expression using bar scatter plots, clearly illustrating these relationships.

We used the Spearman rank correlation coefficient to assess the correlation between transcription factor expression and ATAC peaks, a non-parametric method suitable for evaluating monotonic relationships between variables without assuming a specific distribution. The analysis focused on peaks within 3,000 base pairs upstream and downstream of the target gene promoter region. For each transcription factor, we calculated correlations with all peaks, selecting results with significant correlations (p < 0.01, cor > 0).

Additionally, protein expression data from the TCPA database were used to calculate activity scores for 10 cancer-related pathways based on existing literature. Using the “cor.test” function in R, we computed the Spearman correlation coefficients and p-values between the target gene expression and these pathway activity scores, further exploring the relationship between gene expression and pathway activity.

### CRISP2 gene mutation analysis

In this study, we used CRISPR screening data from the DepMap portal to analyze dependency scores for approximately 17,000 candidate genes using the CERES algorithm. The pan-cancer mutation landscape of the CRISP2 gene was visualized using the “plotmafSummary” function from the “maftools” package in R. To assess the independence between gene expression levels and specific gene mutation types, we employed the “independence_test” function from the “coin” package in R, which utilizes permutation tests to estimate the distribution of the test statistic by randomly rearranging data labels. We evaluated the significance of the relationship between gene mutation types and expression levels using p-values. A significant association was identified when the mutation rate exceeded 10% and the p-value was less than 0.01, followed by visualization. For the TCGA-BRCA project, tumor copy number profiles were analyzed using a GISTIC score-based method. Data from 451 samples were processed to generate a comprehensive CNV profile, with color-coded bar charts illustrating chromosomal copy number changes. Quantitative metrics such as FGA (Fraction of Genome Altered), FGG (Fraction of Genome Gained), and FGL (Fraction of Genome Lost) were defined to measure the total amount of genomic alterations and the extent of gains or losses in clonal regions. To analyze differences among specific gene expression subgroups, we used the ANOVA method, followed by multiple comparisons with the “TukeyHSD” method if ANOVA indicated significance. Scatter plots combined with Spearman rank correlation analysis were used to investigate the correlation between copy number variation (CNV) scores and gene expression levels. The Spearman rank correlation coefficient, a non-parametric method, assesses the monotonic relationship between two variables without assuming a specific data distribution. The experimental data for copy number profiles were obtained from the TCGA Genome Characterization Center through whole-genome microarray measurements. Gene-level copy number estimates were derived using the TCGA FIREHOSE pipeline and the GISTIC2 method. Gene expression differences among various copy number types (−2 to 2) were compared using the Kruskal-Wallis test, a non-parametric method suitable for multiple sample comparisons without assuming a specific data distribution.

### Cell culture and collection of macrophage conditioned medium

RAW 264.7 macrophages (CL-0190) and mouse nucleus pulposus cells (mNPCs) (CP-M146) were obtained from Procell (Wuhan, China). mNPCs were cultured in a specialized medium at 37°C with 5% CO_2_. RAW 264.7 macrophages were maintained in Dulbecco’s Modified Eagle Medium (DMEM) supplemented with 100 U/mL penicillin, 100 mg/mL streptomycin, and 10% fetal bovine serum. To induce a degeneration model, mNPCs were treated with LPS (1 μg/mL), while RAW 264.7 macrophages were polarized into M1 or M2 phenotypes using LPS (1 μg/mL) and interleukin-4 (IL-4) (20 ng/mL), respectively. Various concentrations of Hesperidin (20, 50, and 100 µM) were administered to the experimental groups for 24 and 48 h. When the cell density of RAW 264.7 macrophages or mNPCs reached 60%, LPS or IL-4 was added to the culture medium according to the group requirements, with or without Hesperidin pretreatment. After 24 h, the medium containing LPS, IL-4, or Hesperidin (50 μM) was discarded. Macrophages were then treated with fresh medium for another 24 h. The conditioned medium (CM) was collected by centrifugation at 1,000 g and used for subsequent experiments. mNPCs were cultured under normal or LPS-stimulated conditions in a mixed medium (50% specialized medium and 50% CM from different macrophage groups) to conduct additional experiments.

### Cell viability assessment

To evaluate cell viability, NPCs were cultured in 96-well plates. At specified time points, the initial medium was replaced with a 10% Cell Counting Kit-8 (CCK-8) solution. Absorbance was measured at 450 nm to determine cell viability.

### Flow cytometry analysis

ROS was detected using the fluorescent probe DCFH-DA. The intensity of green fluorescence is directly proportional to the level of ROS. Detect intracellular ROS levels by measuring fluorescence intensity using flow cytometry. Collected RAW 264.7 cells in logarithmic growth phase from passaging culture and inoculated them into a 6-well plate at a density of 2 × 10^4^ cells. After 24 h, treat with 20 μM, 50 μM, and 100 μM hesperidin and LPS, while setting up a blank control group. Continue to culture the 6-well plate in a 37°C, 5% CO_2_ cell incubator for 72 h, remove the cell culture medium, collect the cells in a flow cytometer, wash twice with cold PBS, add a fluorescent probe DCFH-DA with a final concentration of 10 μmol/L, and incubate for 30 min in a 37°C, 5% CO2 cell incubator. Wash the cells twice with cold PBS, resuspend them in 500 µL PBS, and detect them using flow cytometry.

### Reverse transcription-quantitative polymerase chain reaction (RT-qPCR)

For the RT-qPCR analysis, RAW 264.7 macrophages and mNPCs were lysed in Trizol (Invitrogen, CA, United States) to extract total RNA. The RNA was then reverse-transcribed into cDNA using the PrimeScript RT Reagent Kit (TaKaRa Bio, Otsu, Japan). RT-qPCR was conducted using SYBR Green Master Mix (Vazyme, Nanjing, China) along with specific forward and reverse primers to quantify the mRNA expression of target genes. The Gapdh gene served as an internal control, and relative expression levels were determined using the 2^−ΔΔCt^ method.

### Immunofluorescence staining

RAW 264.7 macrophages and NPCs were treated with LPS, IL-4, or Hesperidin in 24-well plates. Cells were fixed with 5% formaldehyde for 15 min and permeabilized with 0.1% Triton X-100 for 10 min. Blocking was carried out with 1% BSA at 37°C for 1 h. Following this, cells were incubated with primary antibodies at 37°C for 4 h, then with Alexa Fluor-594 or Alexa Fluor-488 conjugated secondary antibodies (Abcam) or phalloidin (Beyotime) in the dark at 37°C for 1 h. Nuclei were stained with 4′,6-diamidino-2-phenylindole (DAPI) for 3 min. Images were captured using a fluorescence microscope.

### Statistical analysis

Statistical analyses were performed using R or Python. Each experiment was conducted independently at least three times. Continuous variables are reported as mean ± standard deviation (SD), while categorical variables are represented as proportions. The two-tailed Student’s t-test or Mann-Whitney U test was used to compare the means or distributions of continuous variables. For comparisons involving three or more groups, one-way ANOVA was employed. Statistical significance was set at a p-value of less than 0.05. Categorical variables were analyzed using the χ^2^ test or Fisher’s exact test, depending on the context, with a p-value <0.05 considered significant.

## Results

### Differential gene expression and pathway enrichment in intervertebral disc degeneration

**FIGURE 1:**
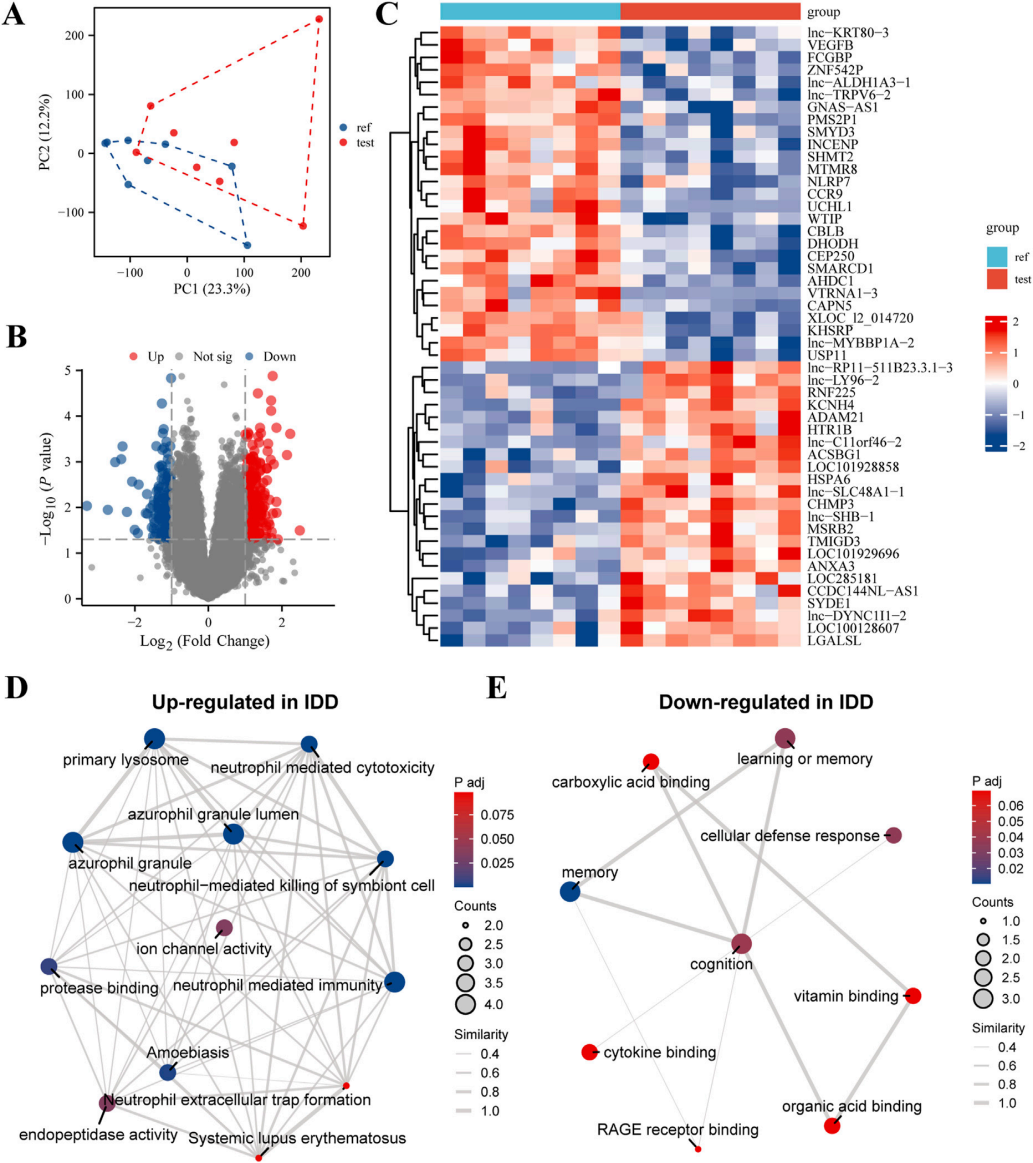
Comprehensive Evaluation of Differential Gene Expression and Enriched Pathways in Intervertebral Disc Degeneration (IDD). **(A)** Principal Component Analysis (PCA) Plot: This plot demonstrates dimensionality reduction of high-dimensional expression data to highlight differences among samples. The x-axis represents the first principal component (PC1), and the y-axis represents the second principal component (PC2). The percentages in parentheses indicate the proportion of variance explained by each principal component. Different sample groups are represented by distinct colors and shapes, allowing for clear visualization of group separations. **(B)** Volcano Plot: This visualization shows the results of differential expression analysis performed using the limma software package. It highlights genes with significant changes in expression levels, showing 30 highly expressed genes (upregulated, red dots) and 30 lowly expressed genes (downregulated, blue dots). The x-axis represents the log2 fold change in gene expression, and the y-axis represents the -log10 adjusted p-value, with a threshold for significance indicated by a horizontal line. **(C)** Heatmap: This heatmap illustrates the expression levels of differentially expressed genes across all samples. Each row corresponds to a gene, and each column corresponds to a sample. The color intensity gradient from blue (indicating low expression) to red (indicating high expression) reflects the level of gene expression. The dendrogram on the left side of the heatmap shows the hierarchical clustering of genes based their expression patterns, which helps to identify groups of genes with similar expression profiles. **(D)** GO/KEGG Enrichment Analysis of Upregulated Genes in IDD: This network visualization presents the results of Gene Ontology (GO) and Kyoto Encyclopedia of Genes and Genomes (KEGG) pathway enrichment analysis for genes that are upregulated in IDD. Nodes in the network represent enriched GO terms or KEGG pathways. The size of each node indicates the number of genes involved, and the color intensity reflects the significance of enrichment (p-value). Connections between nodes indicate shared genes or functional similarities, providing insights into the biological processes and pathways that are activated in IDD. **(E)** GO/KEGG Enrichment Analysis of Downregulated Genes in IDD: Similar to panel **(D)**, this network visualization shows the enriched GO terms and KEGG pathways for genes that are downregulated in IDD. The network provides insights into the functional implications and biological processes affected by the downregulation of genes in IDD, highlighting the pathways and functions that may be compromised.

In our comprehensive analysis of genes associated with IDD, we employed a multilayered bioinformatics approach to elucidate the molecular underpinnings of this condition. Principal Component Analysis (PCA) effectively reduced the dimensionality of high-throughput expression data, revealing distinct sample clusters based on gene expression profiles; the first two principal components accounted for 23.3% and 12.3% of the total variance, respectively (Figure 1A). The differential gene expression analysis, visualized through a volcano plot, identified significantly upregulated and downregulated genes, providing targets for further investigation (Figure 1B). This was complemented by a heatmap, which illustrated the expression patterns across samples with a color gradient, effectively summarizing the data’s complexity (Figure 1C). Further, the biological significance of these expression changes was interrogated using Gene Ontology (GO) and Kyoto Encyclopedia of Genes and Genomes (KEGG) pathway analyses. The upregulated genes in IDD were primarily enriched in immune response and lysosomal pathways, suggesting a potential role in inflammatory processes and cellular waste management (Figure 1D). Conversely, downregulated genes were associated with memory, cognition, and cellular defense mechanisms, indicating a possible link to decreased neural plasticity and altered cellular protection in IDD (Figure 1E). These findings, depicted through GO/KEGG-EMAP visualizations, provided a holistic view of the genetic panorama in IDD, highlighting the complex interplay among various biological processes. Our findings underscore the complexity of IDD pathophysiology and pave the way for targeted therapeutic strategies.

### Gene expression and intersecting pathways in IDD and breast cancer

**FIGURE 2:**
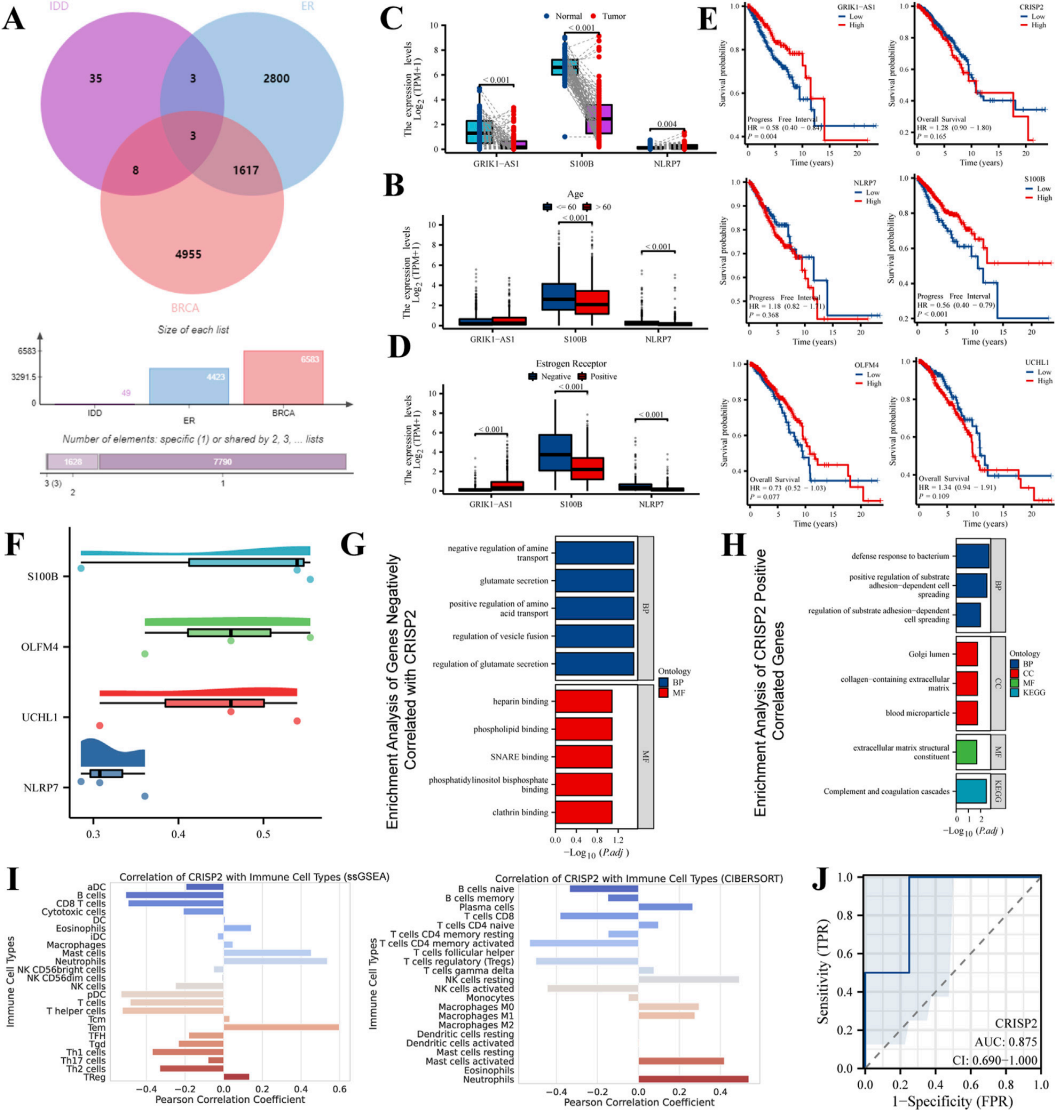
Intersection Analysis and Gene Expression Profiling in IDD and Breast Cancer Subtypes. This figure integrates data extracted from the Gene Expression Omnibus (GEO) database (GSE124272) and RNA sequencing, alongside clinical data from 33 different cancer types obtained from the BRCA-TCGA database. **(A)** Venn Diagram: The Venn diagram illustrates the intersection of differentially expressed genes (DEGs) among IDD, Estrogen Receptor-positive (ER+) breast cancer, and general breast cancer gene lists. The diagram highlights both unique and shared DEGs among these conditions, emphasizing potential molecular links between IDD and breast cancer. **(B)** Boxplot Analysis: The boxplot shows the expression levels of selected DEGs in individuals above and below 60 years of age. This analysis highlights age-associated varieties in gene expression, which may contribute to the pathophysiology of IDD and breast cancer. **(C)** Expression Level Comparison: This panel compares the expression levels of various DEGs in normal versus tumor samples. Statistical significance of the differences is demonstrated, providing insights into the potential roles of these genes in tumor biology. **(D)** Subtype Expression Profile: The expression of selected DEGs is depicted across Estrogen Receptor-negative (ER−) and Estrogen Receptor-positive (ER+) breast cancer subtypes. This comparison highlights subtype-specific gene expression patterns that may inform targeted therapeutic strategies. **(E)** Survival Analysis: Kaplan-Meier survival curves show the association between the expression levels of selected DEGs and patient survival, stratified by high and low expression groups. This analysis is crucial for understanding the prognostic value of these genes in cancer. **(F)** Correlation Plot: This plot presents the Pearson correlation coefficients for selected DEGs with therapeutic outcomes, providing a statistical measure of their potential predictive power in clinical scenarios. **(G)** Enrichment Analysis of Genes Negatively Correlated with CRISP2: This panel displays the enriched biological processes and pathways associated with gene sets negatively correlated with CRISP2 expression. The analysis reveals insights into the biological processes and pathways that these genes may influence, using GO and KEGG enrichment methods. **(H)** Enrichment Analysis of Genes Positively Correlated with CRISP2: Similar to panel **(G)**, this panel shows the enriched biological processes and pathways associated with gene sets positively correlated with CRISP2 expression. Nodes represent specific GO terms or KEGG pathways, and the network visualization indicates the significance and extent of enrichment. **(I)** Immune Cell Correlation: The panel correlates the expression of CRISP2 with various immune cell types, using the CIBERSORT algorithm to assess the cell-type composition. This analysis provides insights into the immune landscape and its potential interactions with gene expression in IDD patients. **(J)** Receiver Operating Characteristic (ROC) Curve: The ROC curve depicts the diagnostic potential of CRISP2 expression in distinguishing between normal and IDD samples. The Area Under the Curve (AUC) provides a quantitative measure of the gene’s discriminative ability.

Our study conducted an in-depth intersection analysis and gene expression profiling for IDD and various breast cancer subtypes, We integrated microarray data from eight healthy volunteers and eight IDD patients from the GEO database (GSE124272), alongside RNA sequencing and clinical data from 33 different cancer types in the BRCA-TCGA database. The Venn diagram (Figure 2A) revealed a significant overlap of differentially expressed genes (DEGs) between IDD, Estrogen Receptor-positive (ER+) breast cancer, and general breast cancer gene lists. This overlap suggests molecular parallels that could shed light on the underlying mechanisms of these diseases. Age-stratified boxplot analyses (Figure 2B) revealed significant differences in the expression levels of selected DEGs, highlighting the influence of age on gene expression dynamics in these conditions. Additionally, we observed distinct expression patterns of specific DEGs when comparing normal to tumor samples, indicating statistically significant discrepancies that may play roles in tumorigenesis (Figure 2C). Our subtype expression profile analysis (Figure 2D) have detailed DEG expression in ER-negative and ER-positive breast cancer subtypes, showcasing subtype-specific gene expression patterns that could inform therapeutic strategies. Kaplan-Meier survival analyses (Figure 2E) established correlations between DEG expression levels and patient survival, emphasizing the prognostic significance of these genes in cancer contexts. Correlation plots (Figure 2F) illustrated Pearson correlation coefficients, suggesting the predictive potential of selected DEGs in relation to clinical outcomes. Enrichment analyses provided a deeper understanding of the biological processes and pathways influenced by gene sets negatively (Figure 2G) and positively (Figure 2H) correlated with CRISP2. Furthermore, the expression of CRISP2 was correlated with various immune cell types (Figure 2I) using the CIBERSORT algorithm to predict cell-type composition, offering insights into the immune response dynamics in IDD patients. Finally, the diagnostic capability of CRISP2 expression in distinguishing between normal and IDD samples was quantified by the Receiver Operating Characteristic (ROC) curve (Figure 2J), with the Area Under the Curve (AUC) providing a robust statistical measure of discriminative performance. These comprehensive analyses reinforce the intricate relationship between gene expression and the pathophysiology of both IDD and breast cancer, while also identifying potential biomarkers for diagnosis and prognosis.

### Comprehensive results of metabolite efficacy and interaction analysis

**FIGURE 3:**
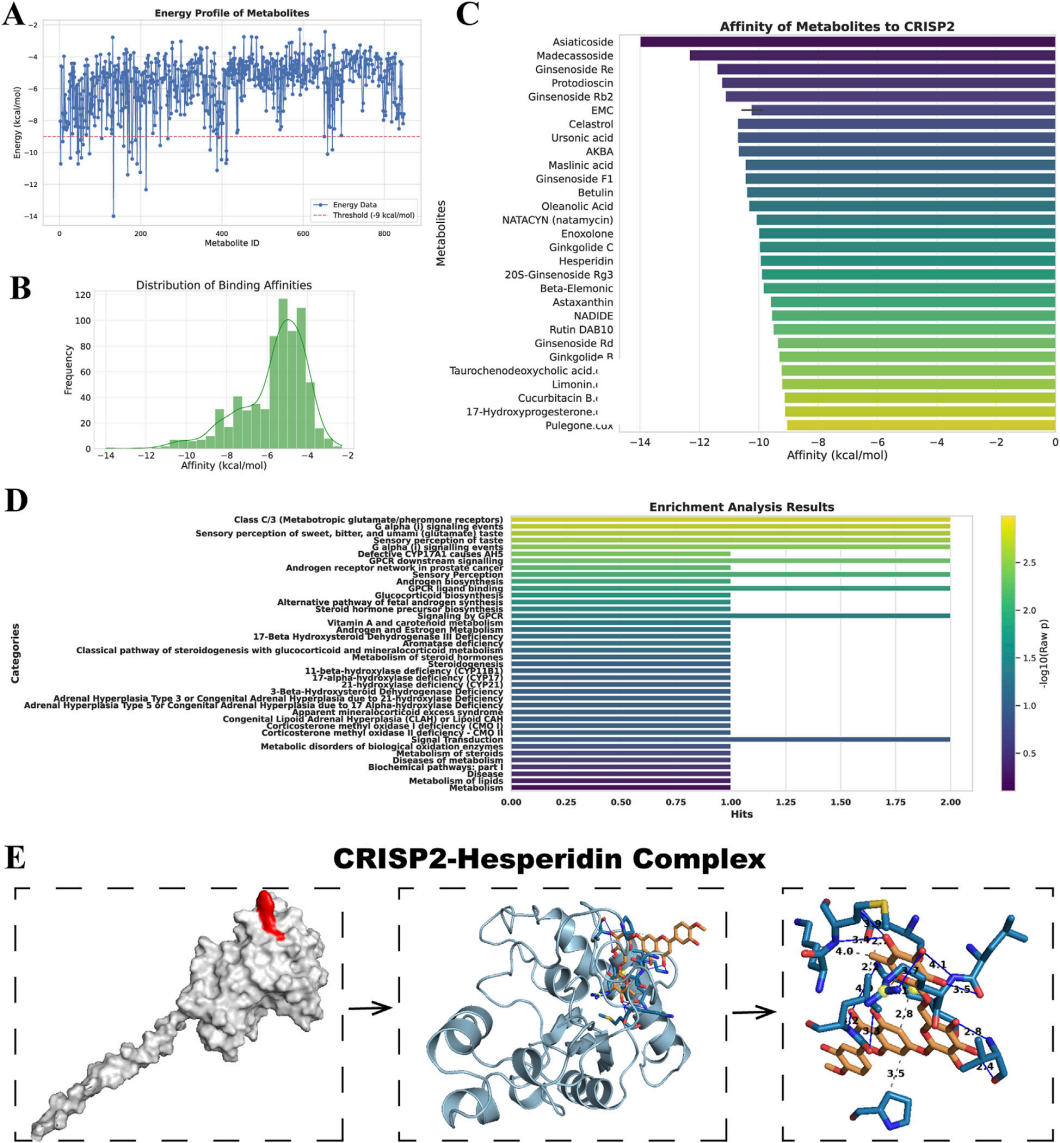
Drug Screening and Metabolite Analysis via Molecular Docking and Enrichment. **(A)** Energy Profile of Metabolites: This panel displays the free energy landscape of metabolites from the MedChemExpress LLC database. Each blue point represents the binding affinity of a small molecule metabolite. The black line overlaid on the plot represents the statistical distribution of these affinities, providing a comprehensive view of the energy profiles. **(B)** Distribution of Binding Affinities: This histogram shows the frequency distribution of the binding affinities among the small molecule metabolites. The plot highlights which binding affinities are most common among the screened metabolites, focusing on those with the most favorable interactions. **(C)** Affinity of Metabolites to CRISP2: In this ranking, selected metabolites are ordered based on their docking affinity to CRISP2 protein, as obtained from the RCSB Protein Data Bank. Higher rankings indicate stronger binding affinities. This analysis identifies potential candidates for further drug development, suggesting which metabolites might effectively target CRISP2. **(D)** Enrichment Analysis Results: This summary highlights the results of the Metabolite Set Enrichment Analysis (MSEA) performed using MetaboAnalyst 6.0. The analysis identifies significant biological pathways impacted by the metabolites under study. **(E)** CRISP2-Hesperidin Complex: This panel visualizes the molecular docking result of Hesperidin with CRISP2, including a detailed interaction map that showcases hydrogen bonds and hydrophobic interactions, processed by PLIP, an automated visualization web service.

In our study, we analyzed a suite of small molecule metabolites for their therapeutic potential. The energy profile (Figure 3A) showed a broad range of binding affinities, with some molecules exhibiting significant binding energy, indicative of potential efficacy. The distribution of these affinities (Figure 3B) highlighted a subset of metabolites with particularly favorable interactions, suggesting strong binding capabilities. Ranking these molecules (Figure 3C) identified several high-affinity candidates for CRISP2, a protein implicated in numerous biological functions, which could be promising leads for drug development. Our metabolite set enrichment analysis (Figure 3D) with MetaboAnalyst 6.0 uncovered key pathways influenced by these metabolites, underscoring their biological relevance. Notably, the molecular docking visualization of the CRISP2-Hesperidin complex (Figure 3E) elucidated the specific interactions at the atomic level, validating our computational approach. These findings, derived from comprehensive computational analyses, inform the next steps in therapeutic exploration and development.

### CRISP2 expression and prognostic significance across various cancers

**FIGURE 4:**
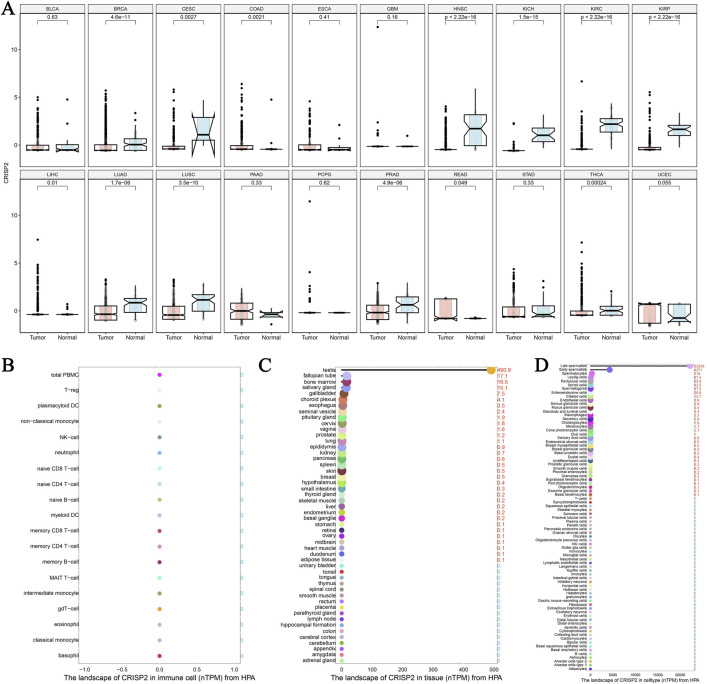
The Landscape of CRISP2 Expression Across Cancers and Tissues **(A)** Differential Expression of CRISP2 Across Various Cancer Types: This plot highlights the significant downregulation of CRISP2 expression in tumors compared to normal tissues across different cancer types. Each plot contrasts CRISP2 expression (z-score normalized) between normal (blue boxplots) and tumor (red boxplots) samples. The x-axis represents different cancer types, while the y-axis shows the z-score normalized expression levels. Statistical significance is indicated by p-values on top of each plot. **(B)** CRISP2 Expression in Immune Cells from the Human Protein Atlas (HPA) Database: This plot displays the normalized transcript per million (TPM) values for CRISP2 across various immune cell types. The x-axis lists the immune cell types, and the y-axis represents the TPM values. **(C)** CRISP2 Expression in Different Tissues from the HPA Database: This plot presents the nTPM (normalized TPM) values for CRISP2 across diverse tissue types. The x-axis lists the tissue types, while the y-axis shows the TPM values. **(D)** CRISP2 Expression in Various Cell Types from the HPA Database: This plot illustrates the nTPM values for CRISP2 across different cell types. The x-axis represents the cell types, and the y-axis demonstrates the nTPM values.

**FIGURE 5:**
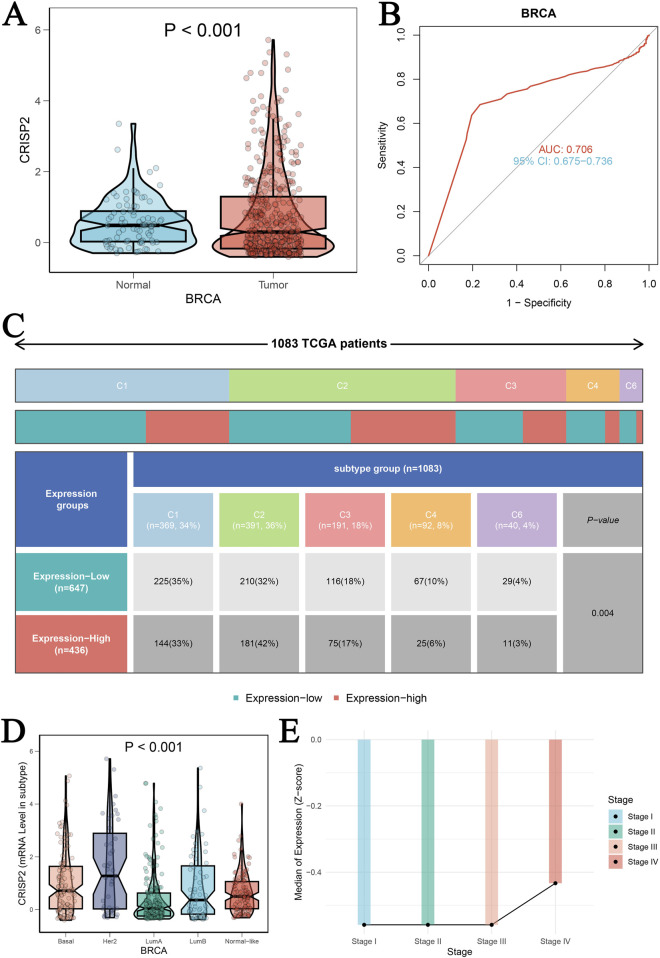
Clinical Prognostic Significance of CRISP2 in BRCA **(A)** Expression Levels of CRISP2 in Normal and Tumor Tissues: This panel presents the expression levels of the CRISP2 gene in normal tissues and BRCA tumor tissues. The data are represented as violin plots with superimposed box plots. Statistical analysis was performed using the t-test, with the significance level indicated (P < 0.001). **(B)** Receiver Operating Characteristic (ROC) Curve: This ROC curve evaluates the diagnostic efficacy of CRISP2 expression in distinguishing between tumor and normal groups. The area under the curve (AUC) is 0.706, with a 95% confidence interval (CI) of 0.675–0.736, indicating moderate diagnostic accuracy. **(C)** Distribution of CRISP2 Expression Across Different Immune Subtypes in the TCGA Cohort: This figure shows the distribution of patients (n = 1,083) in high and low CRISP2 expression groups across six immune subtypes (C1 to C6). A Chi-square test was used to determine the statistical significance of differences among subtypes (P = 0.004). **(D)** Expression of CRISP2 Gene in Different Molecular Subtypes of BRCA: This panel displays the expression levels of the CRISP2 gene across different molecular subtypes of BRCA (Basal, Her2, LumA, LumB) and normal-like tissues. The data are presented as violin plots with box plots. Statistical analysis was performed using ANOVA (P < 0.001). **(E)** Median Expression Levels of CRISP2 Gene Across Different Stages of BRCA: This line graph depicts the median expression levels of the CRISP2 gene across different stages of BRCA (Stage I to IV). The trend indicates an increase in CRISP2 expression with advancing stages.

**FIGURE 6:**
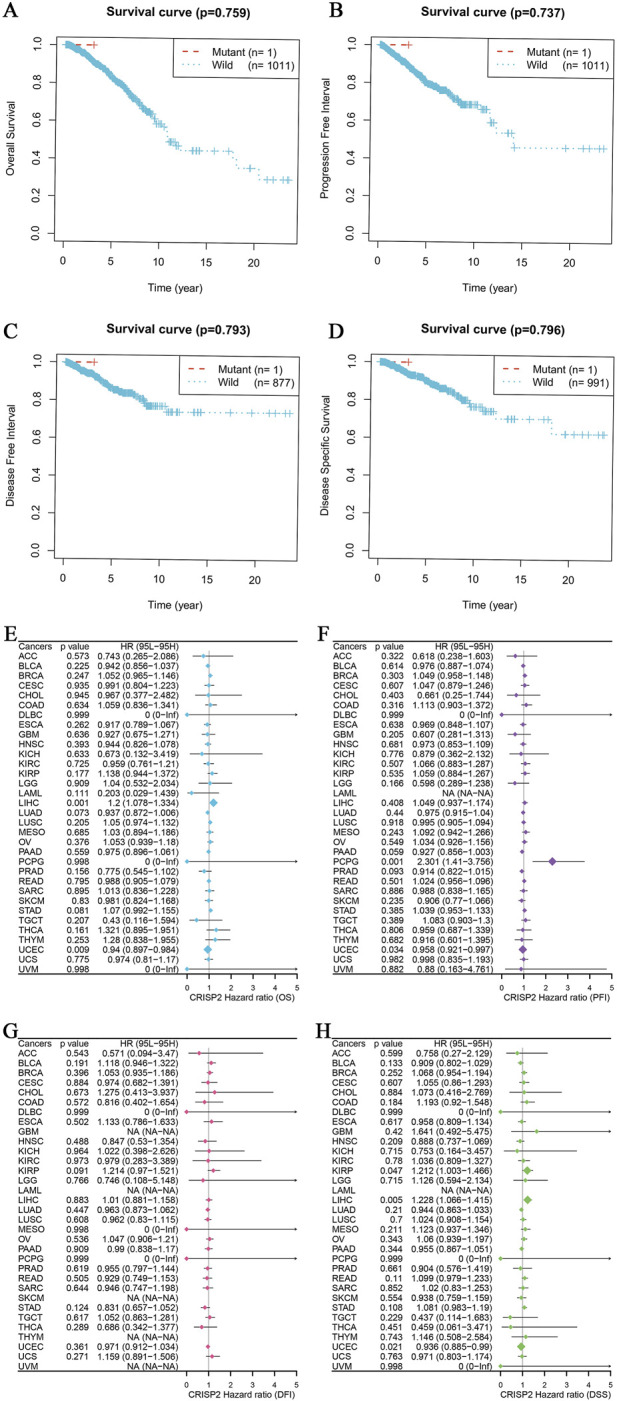
CRISP2 Survival Analysis **(A–D)** Kaplan-Meier Survival Analysis for Four Different Survival Metrics: **(A)** Overall Survival (OS) with p-value = 0.759. The plot compares the survival probabilities over time for patients with mutant (n = 1) and wild-type (n = 1,011) CRISP2. **(B)** Progression-Free Interval (PFI) with p-value = 0.737. The plot shows the progression-free probabilities over time for mutant (n = 1) and wild-type (n = 1,011) CRISP2. **(C)** Disease-Free Interval (DFI) with p-value = 0.793. The plot illustrates the disease-free probabilities over time for mutant (n = 1) and wild-type (n = 877) CRISP2. **(D)** Disease-Specific Survival (DSS) with p-value = 0.796. The plot shows the disease-specific survival probabilities over time for mutant (n = 1) and wild-type (n = 991) CRISP2. **(E–H)** Univariate survival analysis for the same four survival periods: **(E)** Hazard ratios (HR) and 95% confidence intervals (CI) for Overall Survival (OS) across various cancer types. The plot indicates the HRs for mutant versus wild-type CRISP2. **(F)** Hazard ratios (HR) and 95% confidence intervals (CI) for Progression-Free Interval (PFI) across various cancer types. The plot illustrates the HRs for mutant versus wild-type CRISP2. **(G)** Hazard ratios (HR) and 95% confidence intervals (CI) for Disease-Free Interval (DFI) across various cancer types. The plot shows the HRs for mutant versus wild-type CRISP2. **(H)** Hazard ratios (HR) and 95% confidence intervals (CI) for Disease-Specific Survival (DSS) across various cancer types. The plot indicates the HRs for mutant versus wild-type CRISP2. The data points on the plots represent the hazard ratios, while the error bars indicate their 95% confidence intervals. The survival analyses were conducted using patient datasets, with statistical significance assessed using the log-rank test.

The analysis of CRISP2 expression across various cancer types revealed significant downregulation in tumors compared to normal tissues, as demonstrated in Figure 4A. The expression levels of CRISP2 (z-score normalized) were consistently lower in tumor samples (red boxplots) than in normal samples (blue boxplots) across multiple cancer types, with statistical significance indicated by p-values. Further exploration of CRISP2 expression in immune cells, tissues, and cell types from the Human Protein Atlas (HPA) database (Figures 4B–D) showed distinct expression patterns, highlighting its differential expression landscape in the human body. In breast cancer (BRCA), CRISP2 expression was significantly lower in tumor tissues compared to normal tissues (Figure 5A), with diagnostic efficacy indicated by a Receiver Operating Characteristic (ROC) curve showing an AUC of 0.706 (Figure 5B). The distribution of CRISP2 expression across different immune subtypes in the TCGA cohort (n = 1,083 patients) is statistically significant (P = 0.004) (Figure 5C), and its expression varies across BRCA molecular subtypes (P < 0.001) (Figure 5D). The median expression levels of CRISP2 increased with advancing BRCA stages (Figure 5E). Kaplan-Meier survival analyses for Overall Survival (OS), Progression-Free Interval (PFI), Disease-Free Interval (DFI), and Disease-Specific Survival (DSS) showed no significant difference between mutant and wild-type CRISP2 groups (Figures 6A–D). Univariate survival analyses across various cancer types (Figures 6E–H) indicated hazard ratios for mutant versus wild-type CRISP2, underscoring the prognostic relevance of CRISP2 expression. This comprehensive analysis highlights CRISP2’s potential as a diagnostic and prognostic biomarker, warranting further investigation into its functional mechanisms and therapeutic implications.

### Comprehensive analysis of CRISP2 expression in breast cancer

**FIGURE 7:**
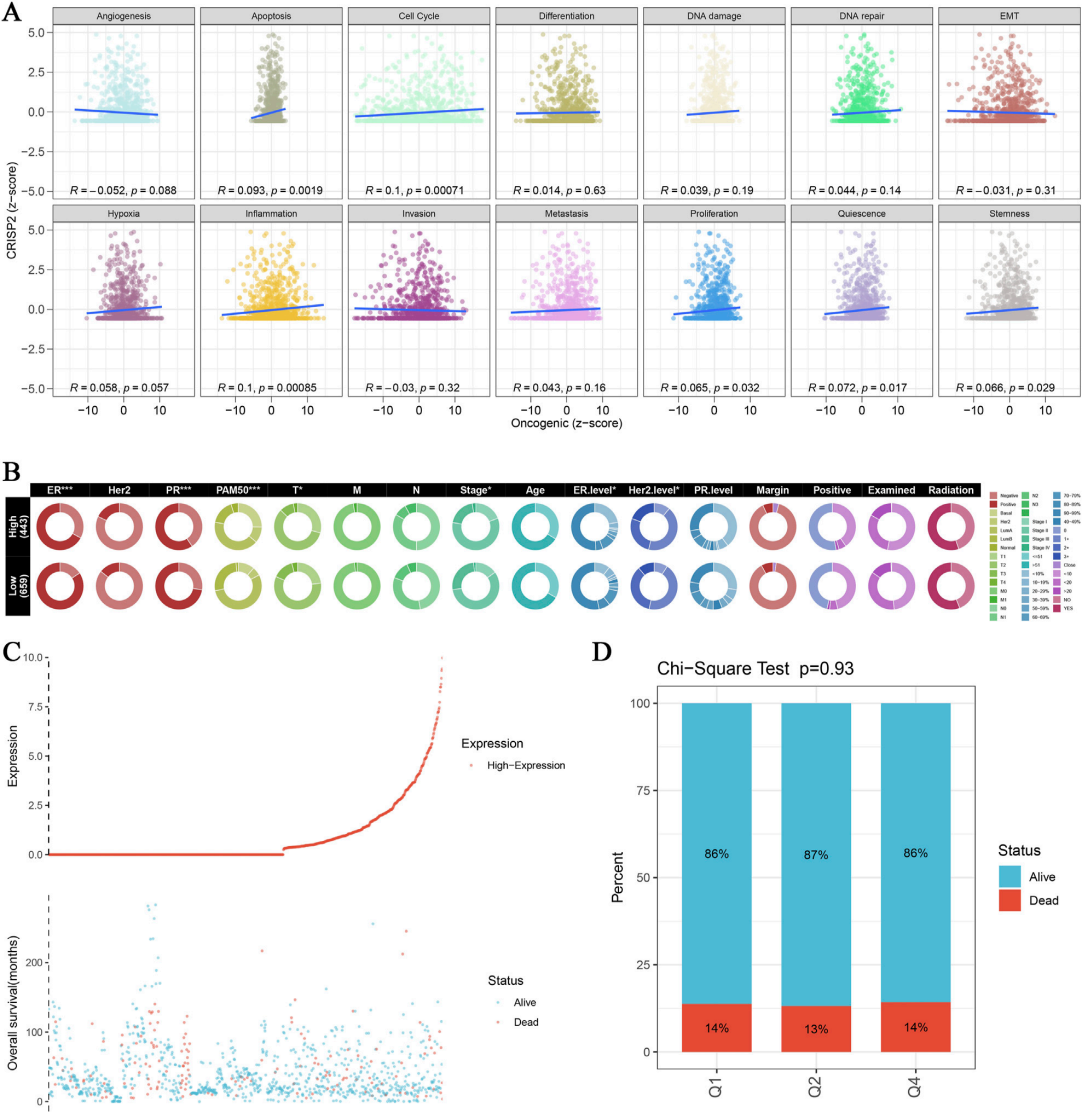
CRISP2 Single Gene GSEA/GSVA Enrichment Analysis **(A)** Gene Set Enrichment Analysis (GSEA): Multiple GSEA were conducted comparing high and low CRISP2 expression groups using the clusterProfiler package. The scatter plots display the correlation between oncogenic scores and various biological processes such as angiogenesis, apoptosis, cell cycle, differentiation, DNA damage, DNA repair, and EMT. Each plot shows the R and p-values indicating the strength and significance of the correlation. **(B)** Chi-square Analysis of CRISP2 Expression and Clinical Traits: Chi-square tests were conducted to analyze the association between CRISP2 expression levels (high vs. low) and various clinical traits. The circular heatmap illustrates the relationship between CRISP2 expression and factors such as gender (sex), PAM50 subtypes, tumor stage, age, ER status, PR status, HER2 status, and treatment types (including margins, positive lymph nodes, radiation, and chemotherapy). Each ring represents a clinical factor with color coding for significance levels. **(C)** Survival Curve Analysis: The survival curve illustrates the relationship between CRISP2 expression and patient survival status (alive vs. dead). The scatter plot at the bottom shows the distribution of overall survival times for patients with different CRISP2 expression levels, highlighting the potential impact of CRISP2 on survival outcomes. **(D)** Quartile Analysis of Survival Status: Chi-square test results comparing the proportion of alive versus dead patients across quartiles (Q1, Q2, Q3) of CRISP2 expression in breast cancer (BRCA) survival. The bar chart indicates that there is no significant difference in survival status across different CRISP2 expression quartiles (p = 0.93).

**FIGURE 8:**
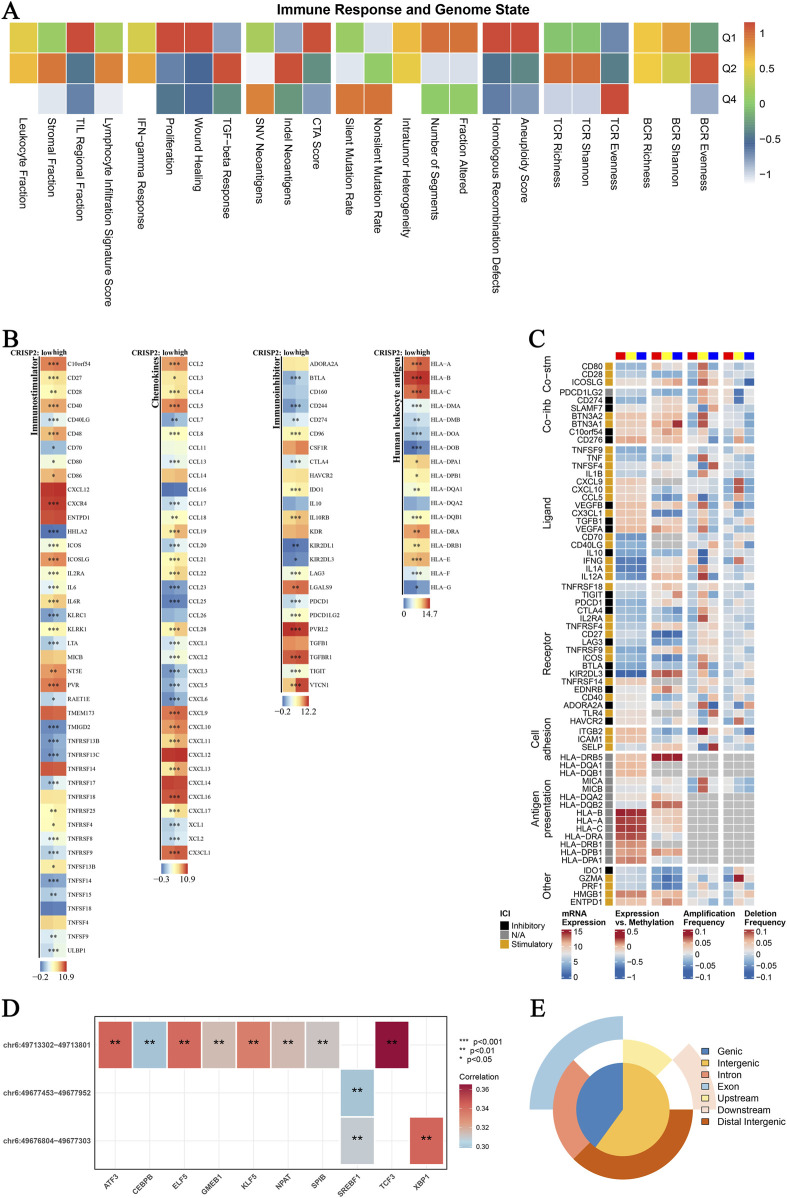
CRISP2 Immune Infiltration Analysis **(A)** Immune Response and Genome State: Heatmap illustrating the correlation between various immune response parameters and genome state. The x-axis represents different immune and genomic features, while the y-axis categorizes the data into quartiles (Q1-Q4) based on the correlation coefficient ranging from −1 to 1. **(B)** CRISP2 Expression in Immunostimulators: Heatmaps showing the expression levels of CRISP2 over diverse immunostimulatory qualities. The three columns speak to distinctive datasets or conditions. Each push compares to a particular quality, and the color scale demonstrates the level of expression or movement, with blue speaking to moo levels and ruddy speaking to all levels. **(C)** Complex Heatmap of Immunomodulators in BRCA: A nitty gritty heatmap appearing the expression and tweak of different immunomodulatory qualities in Breast Cancer (BRCA). Columns speak to diverse qualities categorized into useful bunches (e.g., Co-stim, Lipid, Receptor, Cell Grip). Columns speak to distinctive datasets or test conditions, with the color scale demonstrating the degree of expression, methylation, intensification recurrence, or erasure recurrence. **(D)** Spearman Correlation Analysis between ATAC-Peak and Translation Variables: Heatmap illustrating the Spearman relationship coefficients between ATAC-Peak districts and different translation variables. Each cell within the heatmap speaks to the relationship esteem, with noteworthiness demonstrated by bullets (*p < 0.05, **p < 0.01, ***p < 0.001). The color scale ranges from negative (blue) to positive (red) correlations. **(E)** Types of ATAC-Peak - Visualization with a 3-Venn Pie Chart: Pie chart visualizing the distribution of ATAC-Peak types. Different segments of the chart represent various genomic regions where ATAC-Peaks are located, including genic, intergenic, intron, exon, upstream, downstream, and distal intergenic regions.

In this study, we conducted a comprehensive analysis of CRISP2 expression in relation to various biological processes and clinical traits in breast cancer (BRCA) patients. The Gene Set Enrichment Analysis (GSEA) and Gene Set Variation Analysis (GSVA) comparing high versus low CRISP2 expression groups are presented in Figure 7. These analyses revealed significant associations between CRISP2 expression and processes such as apoptosis and cell cycle, as depicted in Figure 7A. Chi-square tests analyzing the relationship between CRISP2 expression levels and various clinical traits, illustrated in Figure 7B, showed significant associations with factors including gender, PAM50 subtypes, tumor stage, age, ER status, PR status, HER2 status, and treatment types. The survival analysis, presented in Figure 7C, suggested a potential impact of higher CRISP2 expression on patient survival outcomes. However, there was no significant difference in survival status across different CRISP2 expression quartiles, as indicated in Figure 7D (p = 0.93). Furthermore, CRISP2 immune infiltration analysis (Figure 8) highlighted intricate relationships between CRISP2 expression and immune response parameters. Figure 8A provides a heatmap illustrating the correlation between immune response parameters and genome state, while Figure 8B displays heatmaps of CRISP2 expression across different immunostimulatory genes. A detailed heatmap in Figure 8C shows the expression and modulation of various immunomodulatory genes in BRCA. The Spearman correlation analysis between ATAC-Peak regions and various transcription factors, illustrated in Figure 8D, highlight key transcriptional regulators associated with CRISP2. Figure 8E visualizes the types of ATAC-Peak regions using a 3-Venn pie chart, representing various genomic regions where ATAC-Peaks are located. Appropriate statistical methods, including correlation analyses and Chi-square tests, ensured the validity of these findings. These detailed results provide a comprehensive overview of the role of CRISP2 in BRCA, highlighting its potential impact on biological processes, clinical traits, and immune infiltration. This contributes to a deeper understanding of CRISP’2 functional significance in cancer biology.

### CRISP2 gene mutation analysis

**FIGURE 9:**
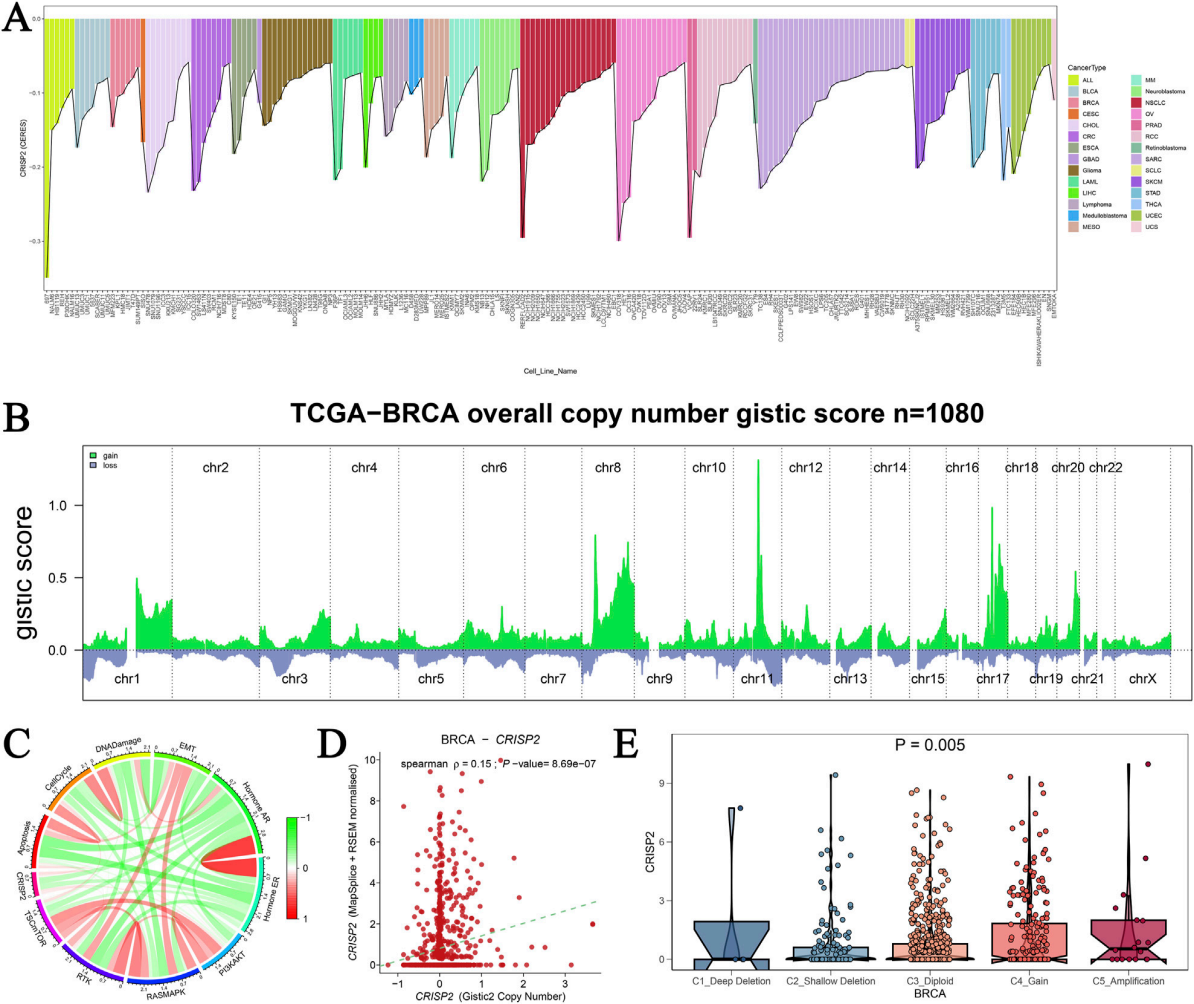
Analysis of CRISP2 Gene Mutation **(A)** CRISP2 Gene Essentiality in Cancer Cell Lines: This panel visualizes the top 200 cell lines from the DepMap database based on CERES scores obtained from a genome-wide CRISPR-Cas9 screen assessing gene essentiality across various cancer cell lines. The bar graph represents the essentiality scores for CRISP2 in different cell lines, highlighting those where CRISP2 is critical for cell survival. **(B)** Copy Number Alterations in Breast Cancer: This graph shows the copy number alterations (CNAs) in breast cancer (BRCA) from the TCGA database, represented as GISTIC2 scores for 1,080 samples. The green areas represent regions of significant amplification, while the blue areas indicate deletions across different chromosomes (chr1 to chrX). **(C)** Correlation Between CRISP2 Expression and Protein Quantification: The circular plot illustrates the correlation between CRISP2 gene expression and functional protein quantification at the pathway level using TCPA-RPPA sequencing data. The connections illustrate significant correlations, with the color intensity representing the strength of the correlation. **(D)** Correlation Between Copy Number Variation and Gene Expression: This scatter plot depicts the Spearman correlation between copy number variation (CNV) scores and CRISP2 gene expression levels. Each point represents an individual sample. Each point represents an individual sample, with a significant positive correlation (p < 0.001) observed. **(E)** CRISP2 Expression Across Different Copy Number Variation Types: Violin plots compare CRISP2 gene expression across different types of copy number variations: deep deletion, shallow deletion, normal, gain, and amplification. The statistical significance of differences in expression levels among the groups is indicated (P = 0.005).

Our analysis of CRISP2 gene mutations underscores its critical role in cancer cell viability, particularly in breast cancer (BRCA). Visualization of the top 200 cell lines from the DepMap database (Figure 9A) shows the essentiality scores for CRISP2, highlighting its importance for cell survival in specific cancer types. Copy number alterations (CNAs) in BRCA from the TCGA database (Figure 9B) demonstrate significant amplifications and deletions across various chromosomes, emphasizing the genomic instability in BRCA and its impact on CRISP2. A circular plot (Figure 9C) correlates CRISP2 gene expression with functional protein quantification at the pathway level using TCPA-RPPA sequencing data, illustrating significant correlations with varying intensity. The scatter plot in Figure 9D shows a significant positive correlation (p < 0.001) between copy number variation scores and CRISP2 gene expression levels, indicating that copy number variations directly influence CRISP2 expression. Violin plots (Figure 9E) compare CRISP2 gene expression across different types of copy number variations (deep deletion, shallow deletion, normal, gain, and amplification), revealing that amplifications lead to higher expression levels (P = 0.005). These comprehensive analyses underscore the potential of CRISP2 as a therapeutic target, with variations in its copy number and expression significantly affecting cellular functions in BRCA.

### Hesperidin reduces the expression of CRISP2, iNOS, and COX2 in IDD, decreases ROS and apoptosis, and lowers inflammatory markers

**FIGURE 10:**
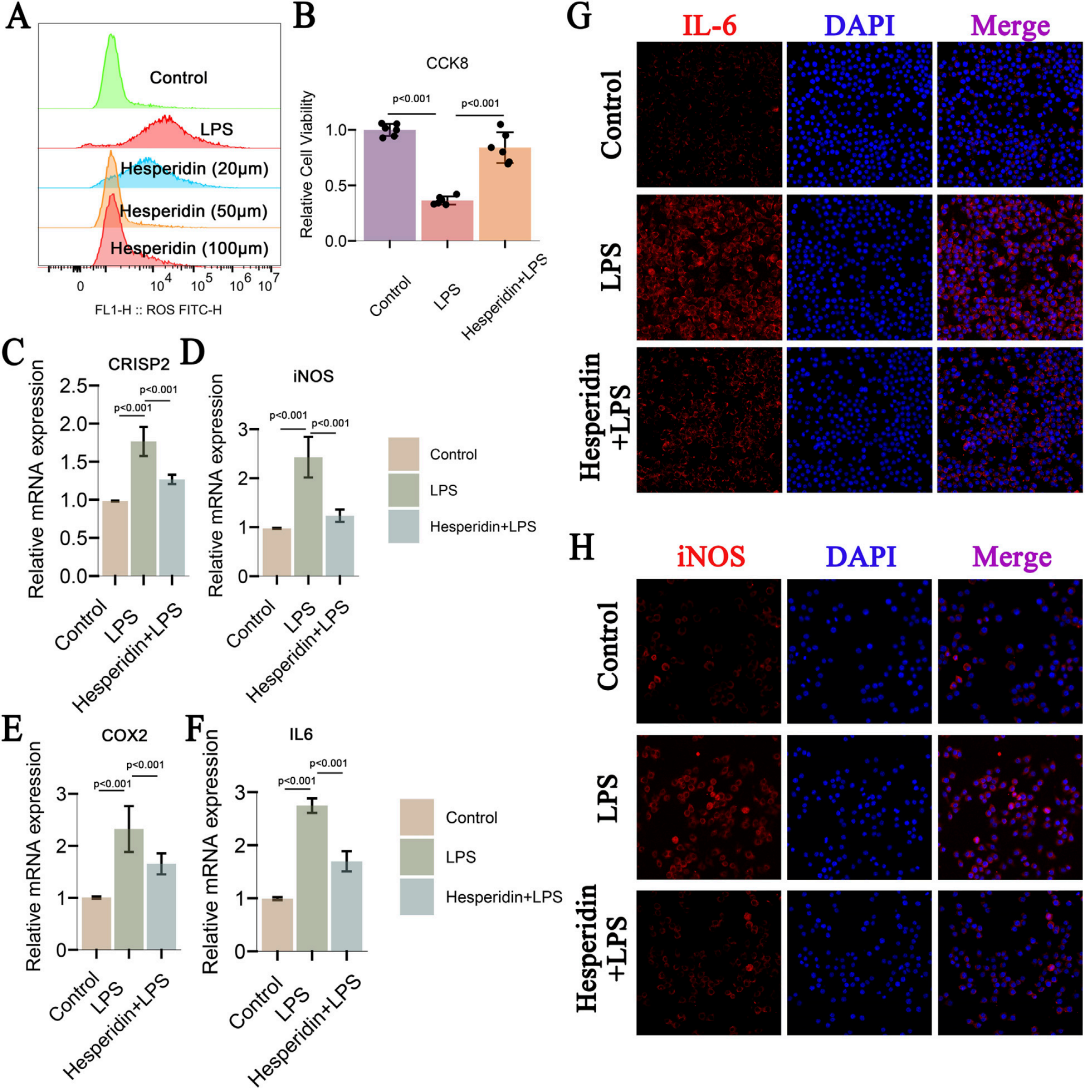
Hesperidin Effects on CRISP2, iNOS, and COX2 in IDD, Decreases ROS and Apoptosis, and Inflammatory Markers **(A)** Flow Cytometry Analysis of ROS Levels: This panel shows flow cytometry analysis of reactive oxygen species (ROS) levels in RAW 264.7 cells treated under different conditions: control, LPS, and Hesperidin at concentrations of 20 μM, 50 μM, and 100 μM. The ROS levels are significantly increased in the LPS group compared to the control, and treatment with Hesperidin at different concentrations reduces ROS levels in a dose-dependent manner. **(B)** Cell Viability Assay (CCK-8): The cell viability assay demonstrates the effects of Hesperidin. Hesperidin treatment significantly improves cell viability compared to the LPS group, indicating a protective effect against LPS-induced cytotoxicity. **(C–F)** Relative mRNA Expression Levels of CRISP2, iNOS, COX2, and IL-6: These panels present the relative mRNA expression levels of CRISP2, iNOS, COX2, and IL-6 in nucleus pulposus cells (NPCs) measured by qRT-PCR. LPS treatment increases the expression of these inflammatory markers, while Hesperidin treatment significantly reduces their expression levels, indicating its anti-inflammatory effects. **(G)** Immunofluorescence Staining of IL-6: This panel shows immunofluorescence staining of IL-6 in Crude 264.7 cells. The cells are recolored for IL-6 (ruddy) and DAPI (blue) for atomic recoloring. The blended pictures appear that IL-6 expression is expanded within the LPS gathered and diminished after Hesperidin treatment. **(H)** Immunofluorescence Staining of iNOS: This panel displays immunofluorescence staining of iNOS in Crude 264.7 cells. The cells are recolored for iNOS (ruddy) and DAPI (blue). The merged images outline that iNOS expression is lifted within the LPS gather and altogether diminished after Hesperidin treatment.

This study investigated the effects of hesperidin on inflammatory responses in IDD using RAW 264.7 cells. Flow cytometry analysis (Figure 10A) revealed that ROS levels significantly increased in the LPS group compared to the control group. However, treatment with hesperidin at concentrations of 20 μM, 50 μM, and 100 μM reduced ROS levels in a dose-dependent manner, indicating its antioxidative properties. Cell viability, assessed using the CCK-8 assay (Figure 10B), showed that LPS exposure significantly decreased cell viability. In contrast, hesperidin treatment at various concentrations notably improved cell viability, suggesting a protective effect against LPS-induced cytotoxicity. Quantitative RT-PCR analysis of nucleus pulposus cells (NPCs) demonstrated that LPS treatment increased the mRNA expression levels of inflammatory markers CRISP2, iNOS, COX2, and IL-6 (Figures 10C–F). Hesperidin treatment significantly reduced the expression levels of these markers, indicating its potent anti-inflammatory effects in NPCs. Immunofluorescence staining was performed to visualize the expression of IL-6 and iNOS in RAW 264.7 cells. The staining for IL-6 (Figure 10G) showed increased expression in the LPS group, which was markedly reduced upon hesperidin treatment. Similarly, iNOS expression (Figure 10H) was elevated in the LPS group and significantly decreased with hesperidin treatment, further confirming the anti-inflammatory properties of hesperidin. These results together suggest that hesperidin effectively mitigates LPS-caused oxidative stress, apoptosis, and inflammatory responses in RAW 264.7 cells, highlighting its potential therapeutic role in managing IDD.

## Discussion

Intervertebral disc degeneration (IDD) is a multifactorial process involving oxidative stress, inflammation, and cellular apoptosis. In our study, we demonstrate that Hesperidin, a natural metabolic compound, effectively mimics the protective effects of estrogen on IDD while mitigating the risk of tumor progression. Hesperidin significantly decreased the expression of CRISP2, iNOS, and COX2, reduced ROS levels and apoptosis, and diminished inflammatory markers in IDD models. By reducing ROS levels, Hesperidin may attenuate oxidative damage to disc cells and modulate inflammatory responses within the intervertebral disc microenvironment. This anti-oxidative and anti-inflammatory potential of Hesperidin presents a promising avenue for therapeutic intervention in IDD, potentially slowing down or preventing disc degeneration. Our analysis revealed the correlation between CRISP2 expression and immune cell infiltration, with survival analysis indicating that CRISP2 levels were associated with patient outcomes across various cancer types. These findings highlight Hesperidin’s potential as a natural metabolic regulator that synergizes with estrogen therapy to promote spinal health and reduce cancer risks.

Previous research has explored the role of estrogen in maintaining disc integrity and its associated cancer risks (Hu et al., 2012; Liang and Shang, 2013). Estrogen’s interaction with specific receptors is known to play a critical role in maintaining disc health but is associated with increased risks of breast and endometrial cancers (Pearce and Jordan, 2004; Hua et al., 2018). Our study advances this understanding by identifying Hesperidin as a compound that can mimic estrogen’s protective effects on IDD without increasing cancer risk. This aligns with and extends prior research on the role of metabolic dysregulation in IDD pathogenesis and the potential of natural compounds as therapeutic agents (Kochar Kaur, 2022; Khotimchenko et al., 2022). Furthermore, chronic inflammation and oxidative stress are implicated in various cancers, including those affecting the gastrointestinal tract. Hesperidin, through its antioxidant properties, has been studied for its potential chemopreventive effects against certain cancers. While direct evidence linking Hesperidin to cancer prevention in the context of IDD remains limited, its ability to mitigate oxidative stress and inflammation suggests a broader therapeutic potential in reducing cancer risk associated with chronic inflammatory conditions.

CRISP2 has been implicated in various cellular processes, including cell proliferation, apoptosis, and differentiation. Emerging evidence suggests that CRISP2 may play a role in maintaining disc cell homeostasis. Dysregulation of CRISP2 expression has been observed in degenerated disc tissues, indicating its potential involvement in IDD pathogenesis. The precise mechanisms by which CRISP2 influences disc degeneration remains to be fully elucidated, but it is postulated that CRISP2 may interact with signaling pathways that regulate oxidative stress and inflammation. Modulating CRISP2 expression or function may help maintain disc cell homeostasis and prevent degeneration (Cheng et al., 2021; Hermawan et al., 2021). In our study, Hesperidin’s ability to reduce CRISP2 expression was evident through multiple bioinformatics analyses. The use of Bioconductor Limma package enabled the identification of DEGs from the GEO database, confirming Hesperidin’s regulatory impact. Functional enrichment analyses through GO and KEGG further illustrated Hesperidin’s influence on key metabolic pathways associated with IDD and cancer progression. The immunological assessments via CIBERSORT and ssGSEA underscored the compound’s effects on immune cell infiltration, linking CRISP2 to various immune cell types, thus reinforcing the anti-inflammatory and anti-tumorigenic properties of Hesperidin.

The importance of cell death and metabolic regulation in disease progression is increasingly recognized, providing new targets and strategies for drug development (Wang and Zhang, 2024; Liu and Ren, 2023). The study of gene expression and regulatory mechanisms in diseases has been deepening, offering crucial insights into the onset and progression of diseases (Zhao et al., 2024). This study establishes Hesperidin as a promising natural metabolic regulator for IDD treatment, offering synergistic benefits with estrogen therapy without increasing cancer risk. In addition, CRISP2 can become a valuable diagnostic and therapeutic target in IDD. Future studies should focus on validating those findings *in vivo*, exploring the specific molecular mechanisms involved, and developing combination therapies to maximize therapeutic outcomes. These efforts will pave the way for new clinical approaches that improve spinal health and reduce the burden of IDD and its associated complications. By addressing the current limitations and expanding on this study, we can enhance our understanding of IDD and develop more effective, safer treatments for patients.

Despite the promising findings, several limitations should be acknowledged. First, the study primarily relies on bioinformatics analyses and *in vitro* experiments, which may not fully capture the complexity of IDD and its interaction with systemic metabolic processes *in vivo*. Second, the sample size for RNA sequencing and clinical data from various cancers types was limited, potentially affecting the generalizability of the results. Third, while the molecular docking and molecular experiments demonstrated the interaction between Hesperidin and CRISP2, further *in vivo* studies are required to confirm these findings and determine the long-term safety and efficacy of Hesperidin in clinical settings. To address these limitations, further research should include large sample sizes and diverse patient populations to enhance the generalizability of the findings. *In vivo* studies are crucial to validate the therapeutic potential and safety of Hesperidin over extended periods. Additionally, exploring the molecular mechanisms underlying Hesperidin’s effects on immune cell infiltration and tumor progression will provide deeper insights into its therapeutic benefits and risks. Investigating the combination of Hesperidin with other natural compounds or existing treatments may also offer new avenues for enhancing its efficacy and safety profile.

These findings not only advance the clinical understanding of IDD treatment but also provide a foundation for future research into natural metabolic regulators that can synergize with existing therapies to provide holistic, multi-targeted treatment strategies. In the future, it’s essential to consider potential adverse effects, such as Gastrointestinal Effects, Drug Interactions with certain medications, Allergic Reactions, etc. In addition, while acute toxicity of Hesperidin has been low, there is a need for more long-term studies to ascertain its safety profile with extended use, especially at higher doses or in specific patient populations. In conclusion, while Hesperidin shows promise as a therapeutic agent for IDD and potentially as a safer alternative to estrogen therapy, vigilance regarding its potential side effects and interactions is crucial. Continued research and clinical monitoring will be instrumental in fully elucidating its therapeutic benefits and ensuring its safe use in clinical practice. Multiple studies have shown that by improving drug delivery systems and leveraging nanotechnology, drug targeting and therapeutic efficacy can be markedly improved (Chen et al., 2024; Liu et al., 2024; Nittayacharn et al., 2024). In the future, biomaterials based on these mechanisms could demonstrate broad application prospects in various biomedical and engineering fields, as shown in previous studies (Pan et al., 2024; Wu et al., 2024). Diverse patient studies and *in vivo* experiments are essential to confirm these findings and explore the specific molecular mechanisms of Hesperidin’s effects. These studies pave the way for developing new clinical strategies that improve spinal health and reduce the burden of IDD.

## Conclusion

This study demonstrates that Hesperidin, a natural metabolic compound targeting CRISP2, effectively mimics the protective effects of estrogen on IDD while mitigating cancer risks. Hesperidin significantly reduced the expression of CRISP2, iNOS, and COX2, decreased ROS levels, and diminished inflammatory markers in IDD models. These findings highlight Hesperidin’s potential as a natural metabolic regulator that synergizes with estrogen therapy to promote spinal health and reduce cancer risks. The study contributes to the understanding of IDD treatment by integrating bioinformatics and multi-omics approaches, providing a robust theoretical framework for the therapeutic applications of hesperidin.

## Data Availability

The datasets presented in this study can be found in online repositories. The names of the repository/repositories and accession number(s) can be found in the article/Supplementary Material.
