# Supplementary figures and images for: Investigating the therapeutic potential of hesperidin targeting CRISP2 in intervertebral disc degeneration and cancer risk mitigation
Source: Front Pharmacol. 2024 Aug 29;15:1447152. doi: 10.3389/fphar.2024.1447152 (PMC11390660; doi:10.3389/fphar.2024.1447152)

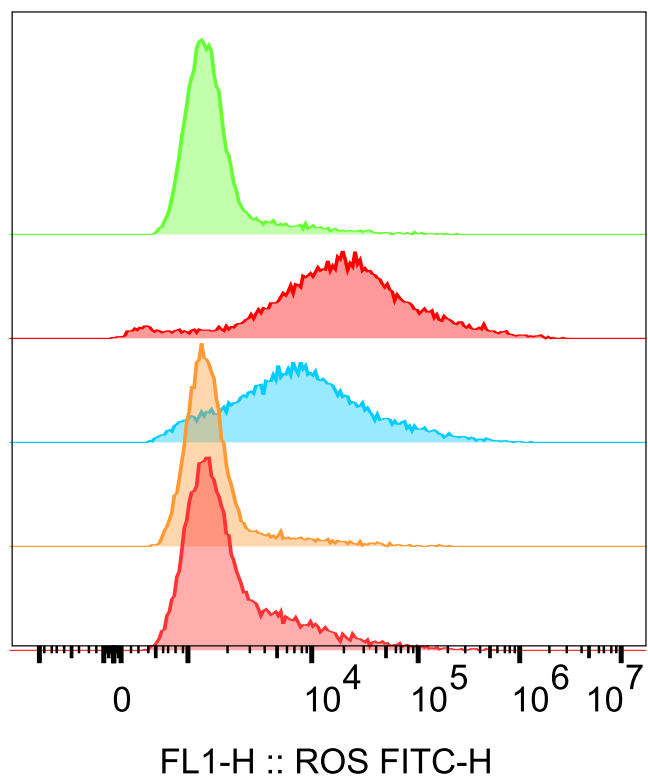

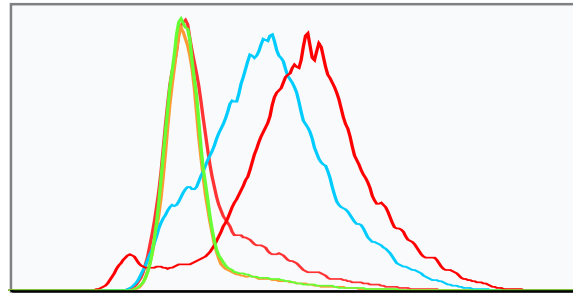

FL1-H :: ROS FITC-H

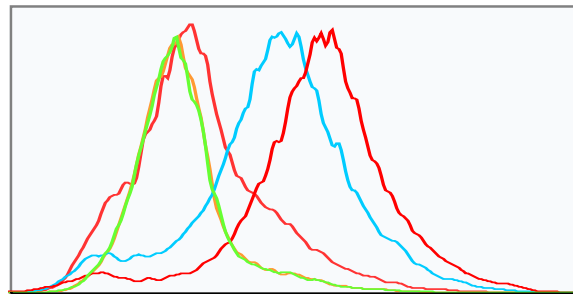

FL1-A :: ROS FITC-A

Supplement: Supplementary file 1 [file DataSheet1.ZIP › rawdata(补充材料上传)/figure10AROS/08-Jun-2024-Layout.pdf]

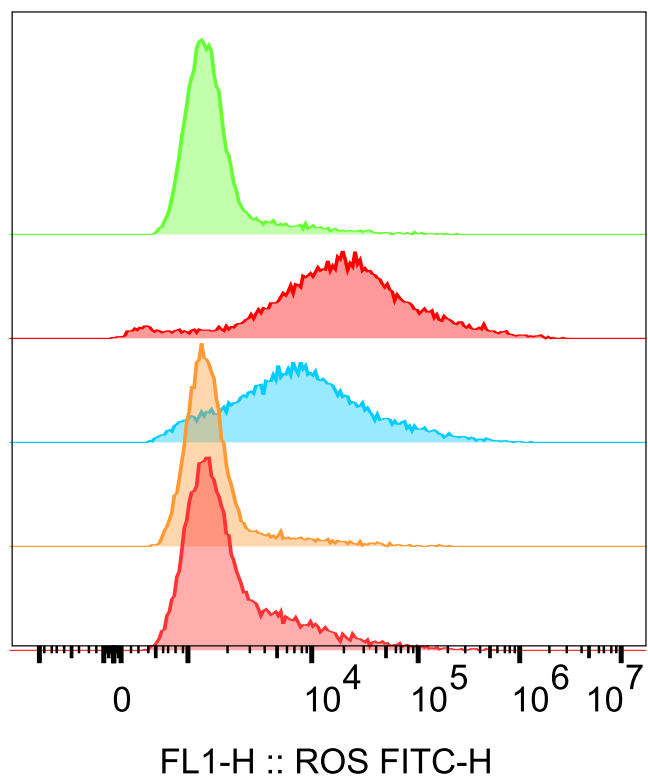

Supplement: Supplementary file 1 [file DataSheet1.ZIP › rawdata(补充材料上传)/figure10AROS/08-Jun-2024-Layout/08-Jun-2024-Layout_1.pdf]

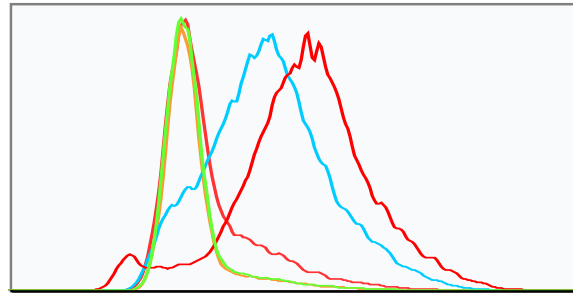

FL1-H :: ROS FITC-H

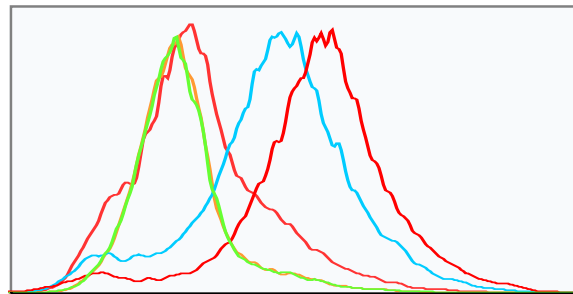

FL1-A :: ROS FITC-A

Supplement: Supplementary file 1 [file DataSheet1.ZIP › rawdata(补充材料上传)/figure10AROS/08-Jun-2024-Layout/08-Jun-2024-Layout_2.pdf]

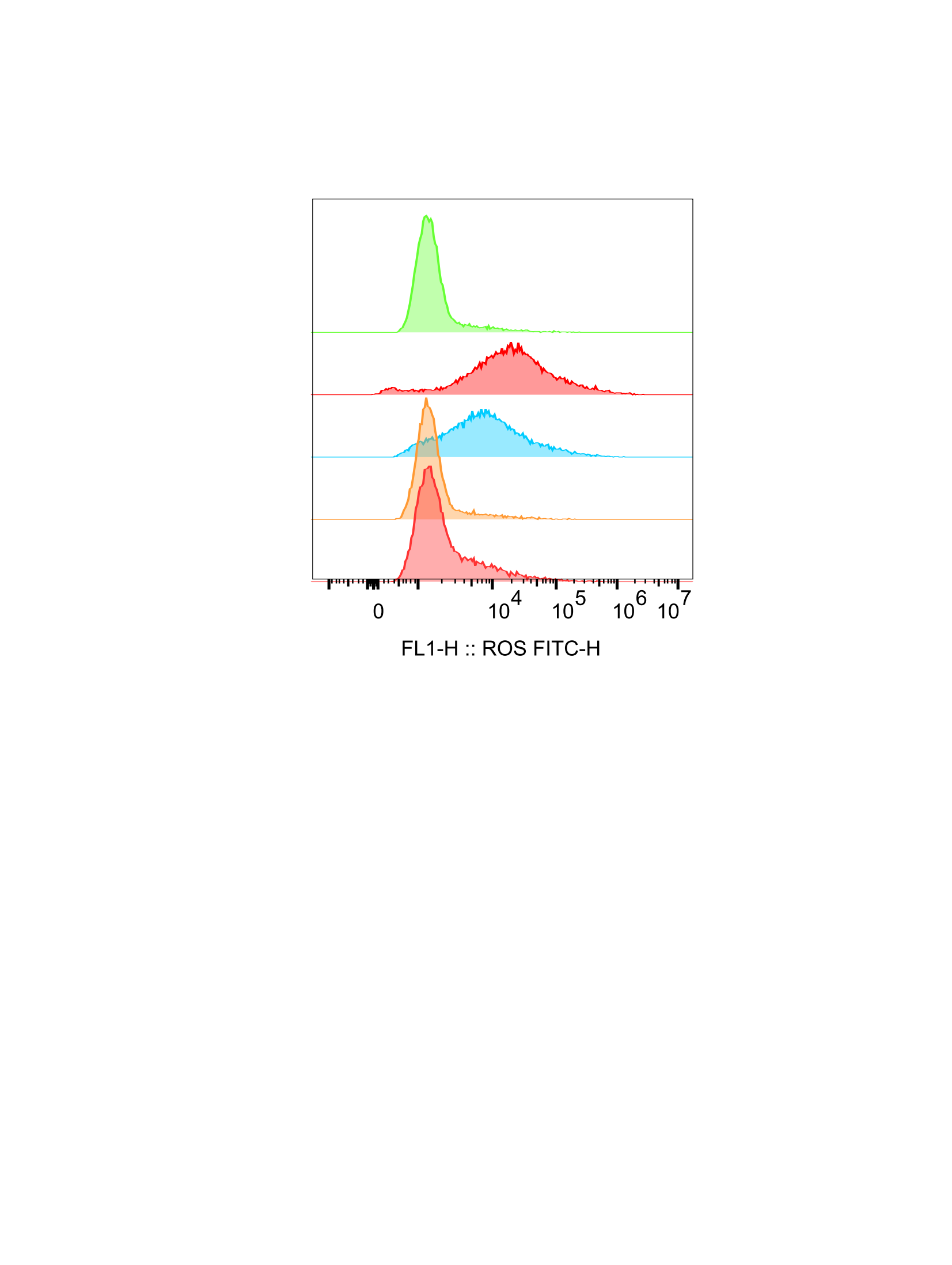

Supplement: Supplementary file 1 [file DataSheet1.ZIP › rawdata(补充材料上传)/figure10AROS/08-Jun-2024-Layout/08-Jun-2024-Layout_1_00.tif]

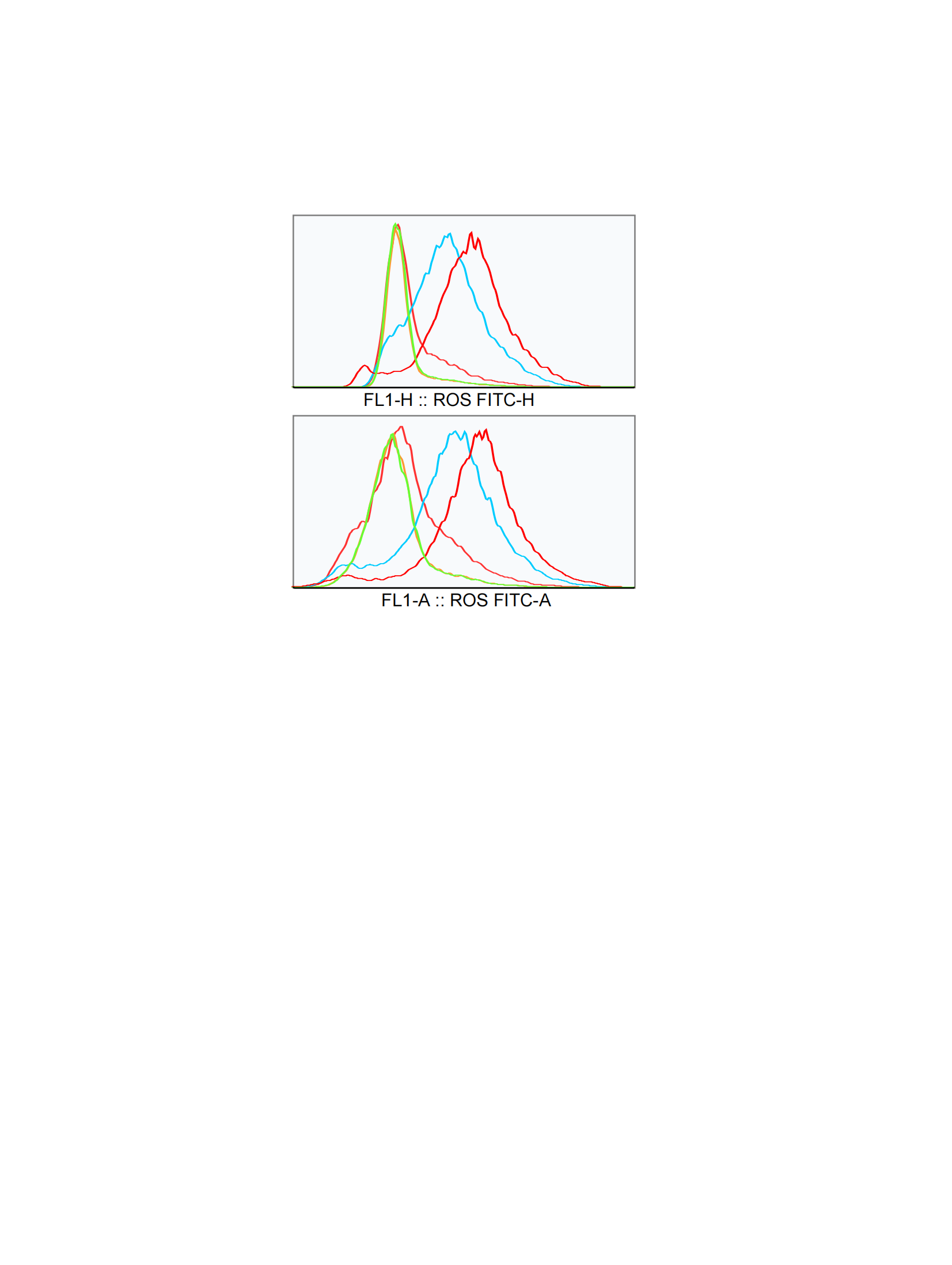

Supplement: Supplementary file 1 [file DataSheet1.ZIP › rawdata(补充材料上传)/figure10AROS/08-Jun-2024-Layout/08-Jun-2024-Layout_2_00.tif]

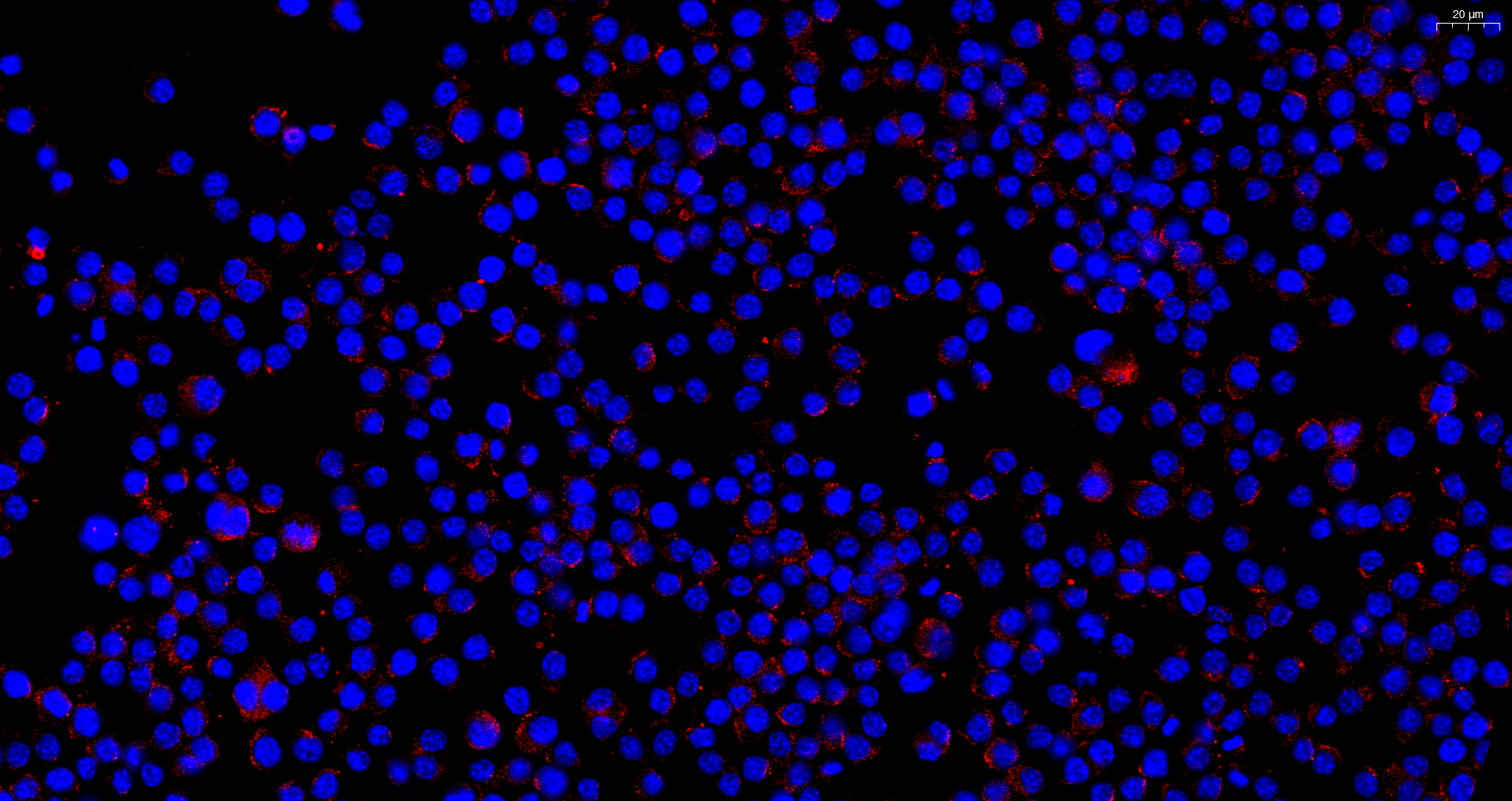

Supplement: Supplementary file 1 [file DataSheet1.ZIP › rawdata(补充材料上传)/figure10G免疫荧光IL6/1.control/RAW IL-6 - Annotation 1_40.0x.tif]

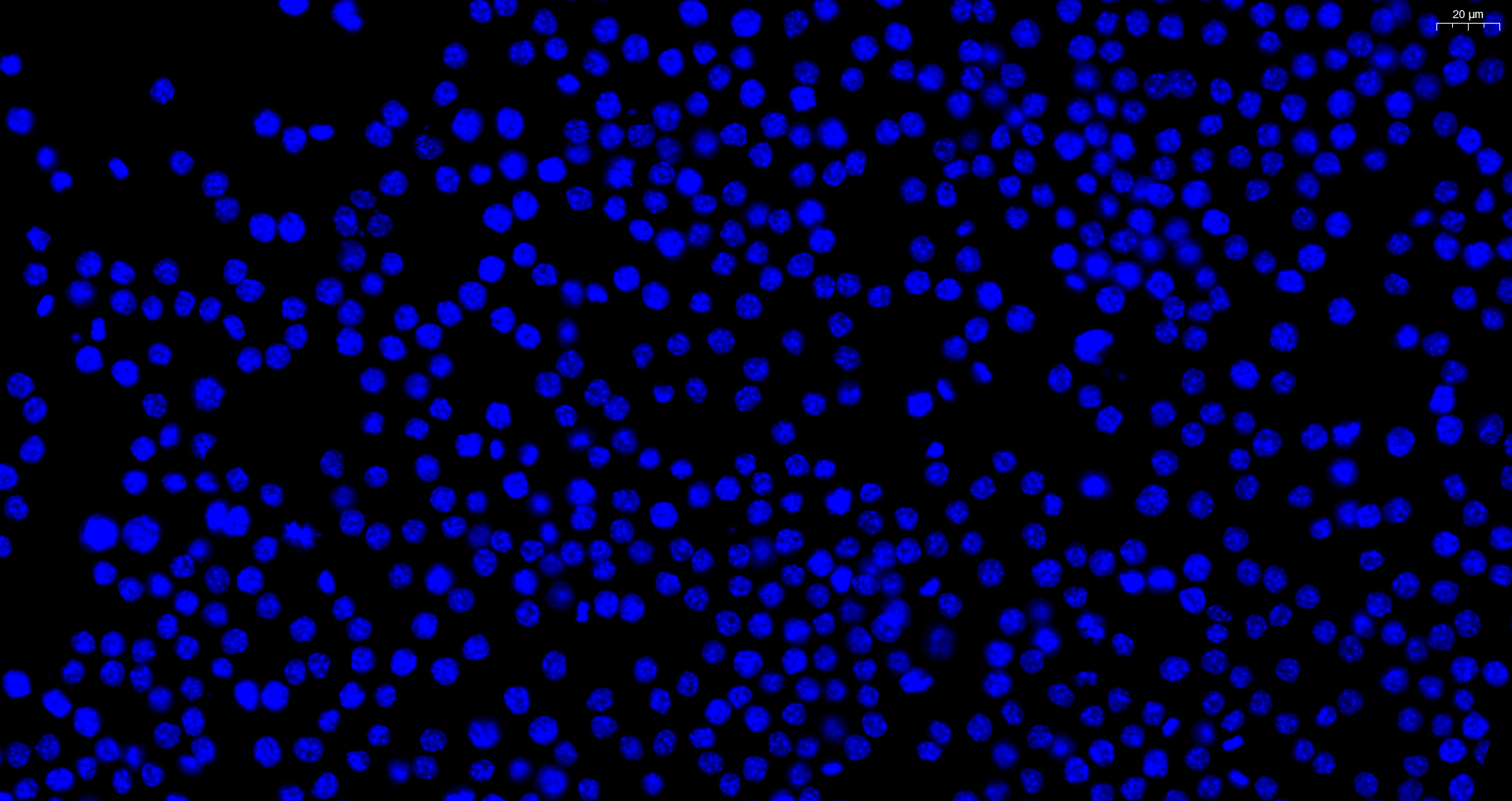

Supplement: Supplementary file 1 [file DataSheet1.ZIP › rawdata(补充材料上传)/figure10G免疫荧光IL6/1.control/DAPI_40.0x.tif]

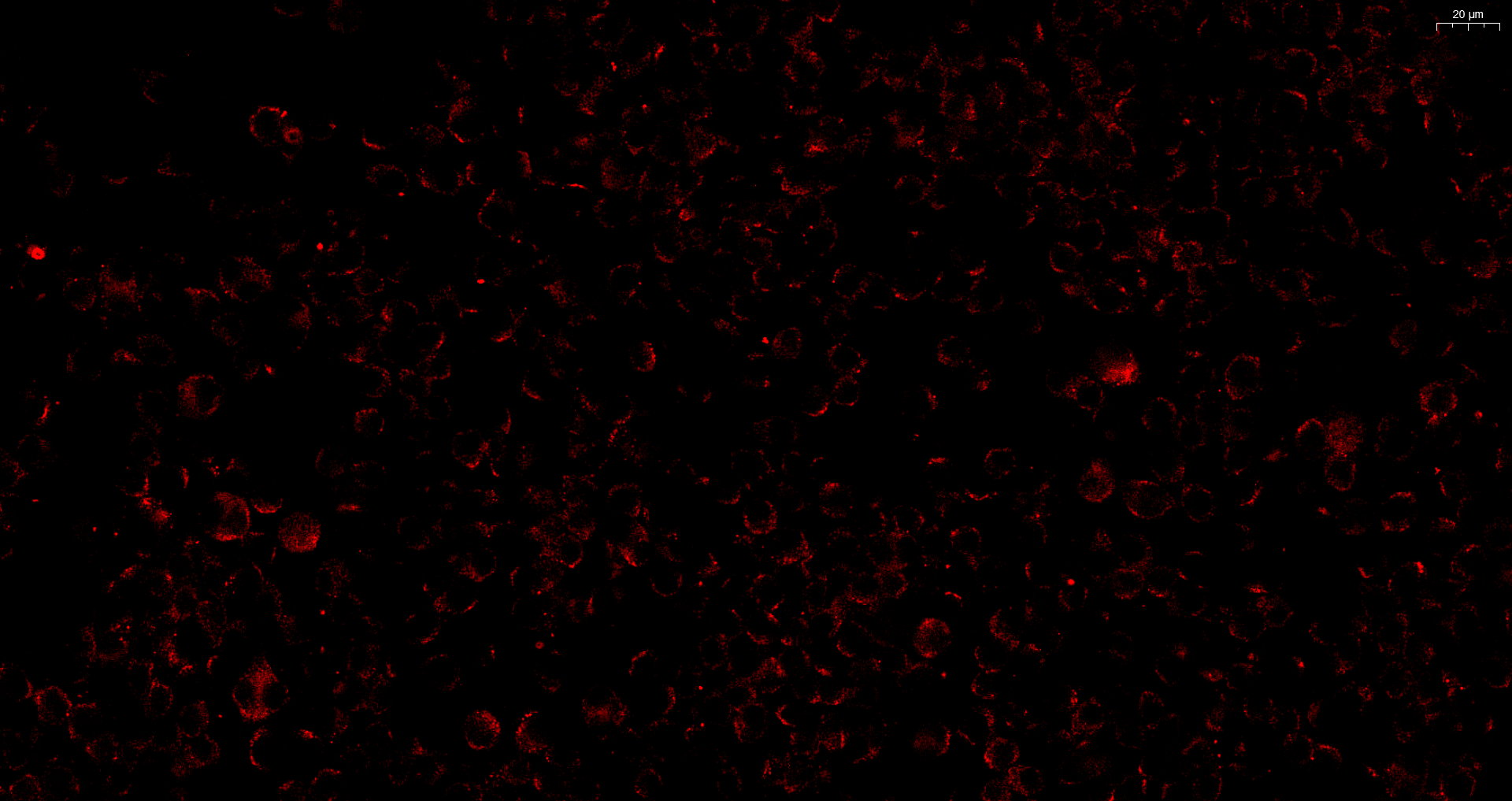

Supplement: Supplementary file 1 [file DataSheet1.ZIP › rawdata(补充材料上传)/figure10G免疫荧光IL6/1.control/IL6_40.0x.tif]

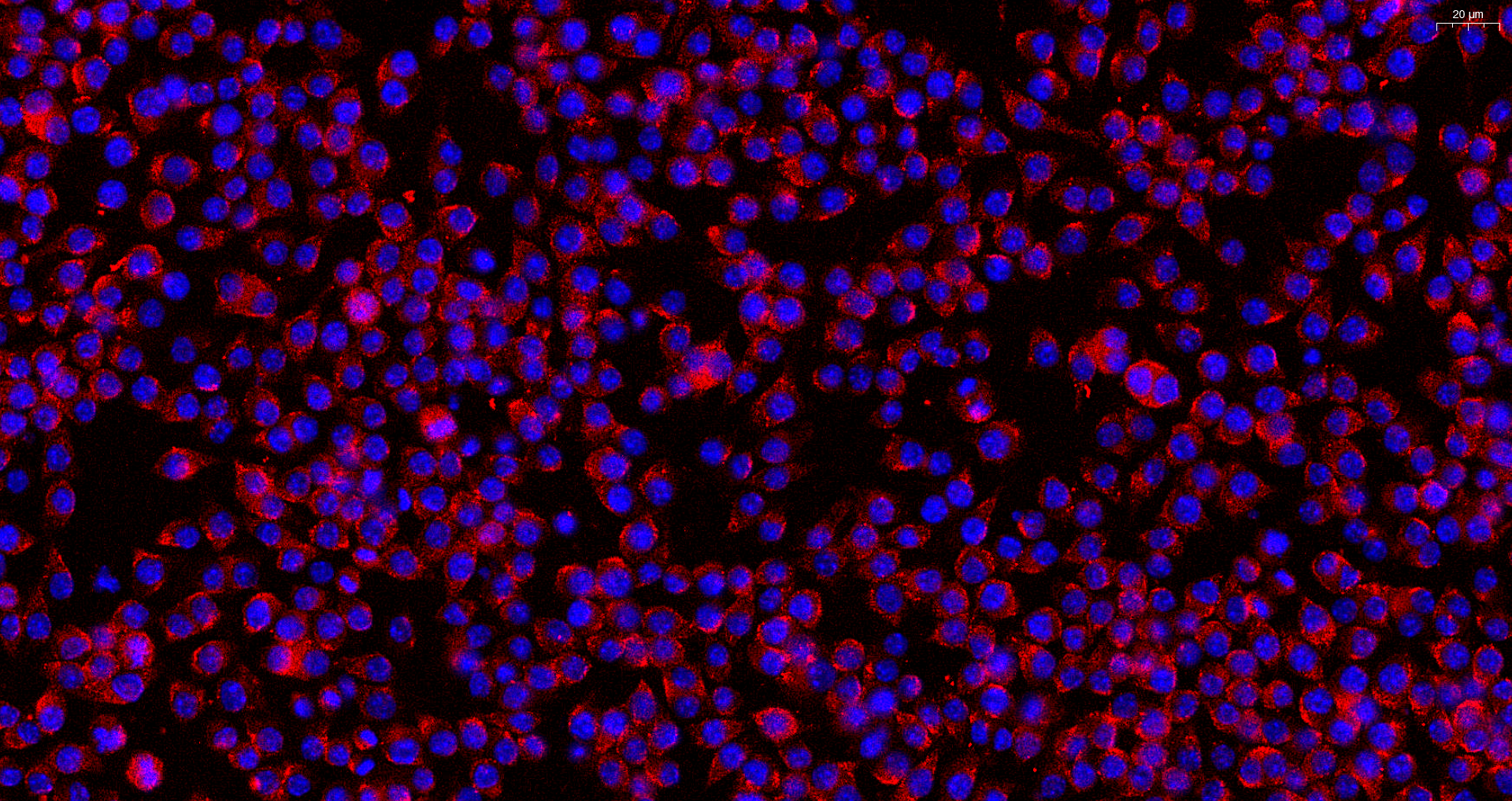

Supplement: Supplementary file 1 [file DataSheet1.ZIP › rawdata(补充材料上传)/figure10G免疫荧光IL6/2.LPS/RAW IL-6 - Annotation 2_40.0x.tif]

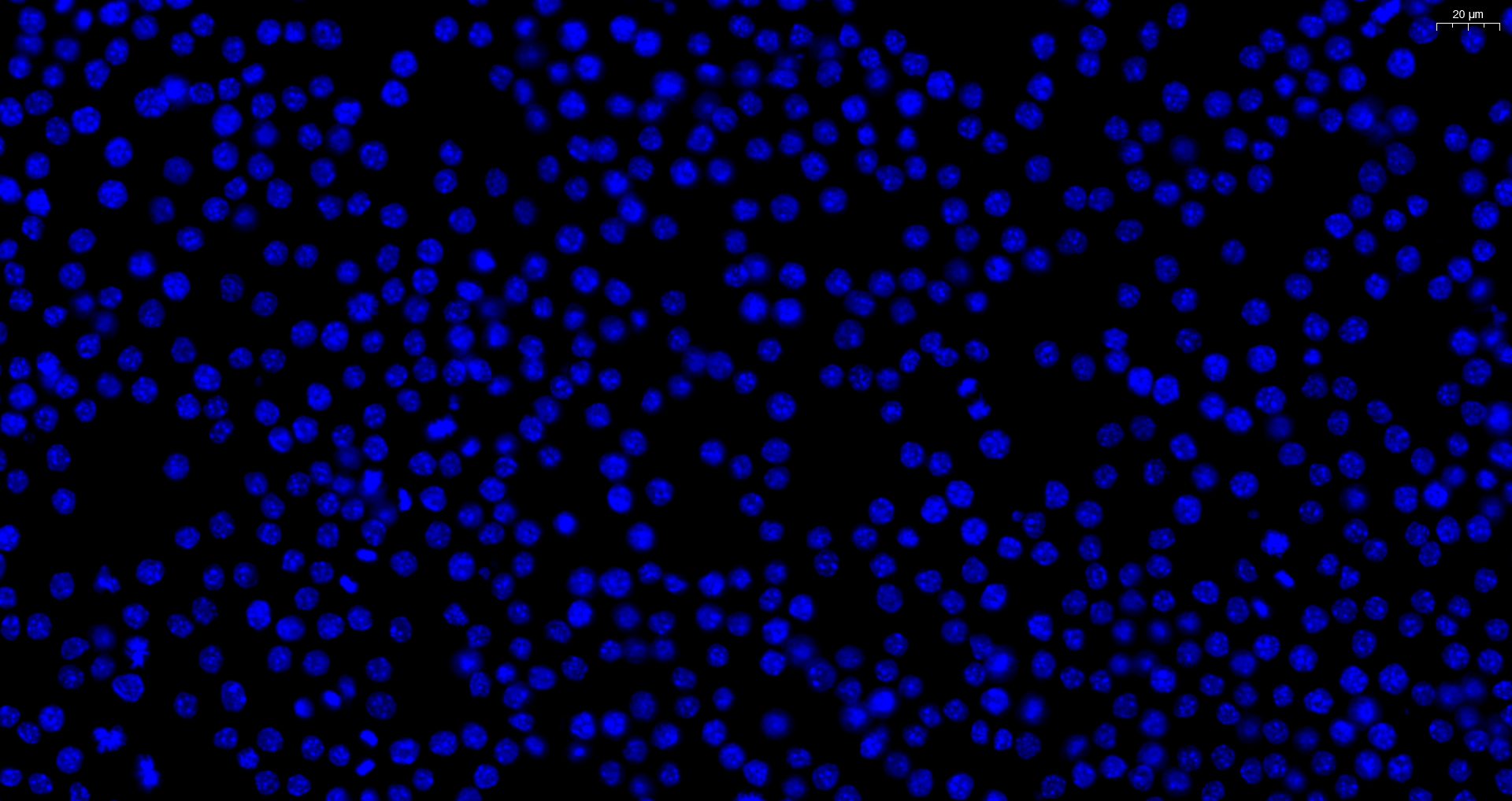

Supplement: Supplementary file 1 [file DataSheet1.ZIP › rawdata(补充材料上传)/figure10G免疫荧光IL6/2.LPS/DAPI_40.0x.tif]

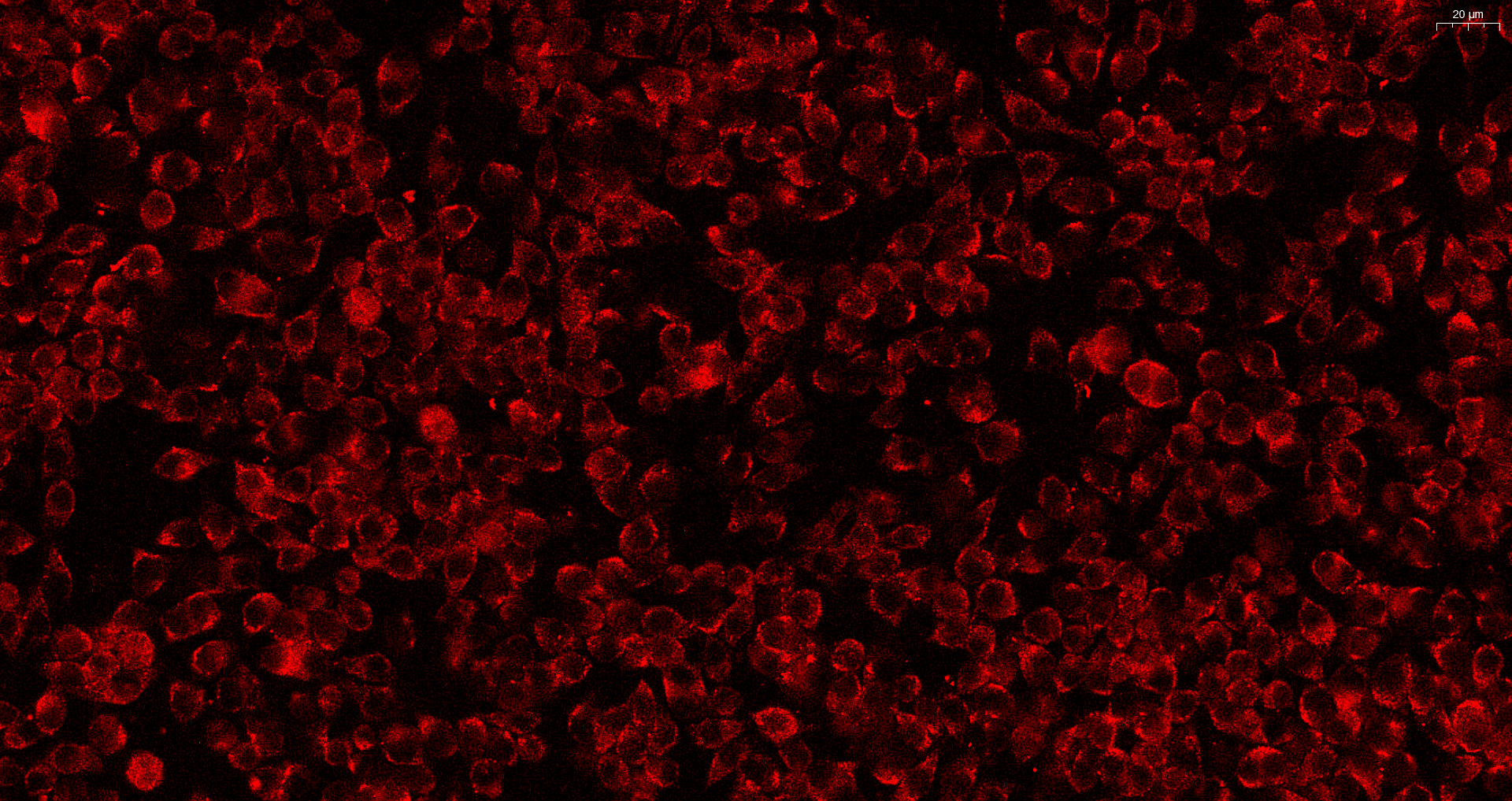

Supplement: Supplementary file 1 [file DataSheet1.ZIP › rawdata(补充材料上传)/figure10G免疫荧光IL6/2.LPS/IL6_40.0x.tif]

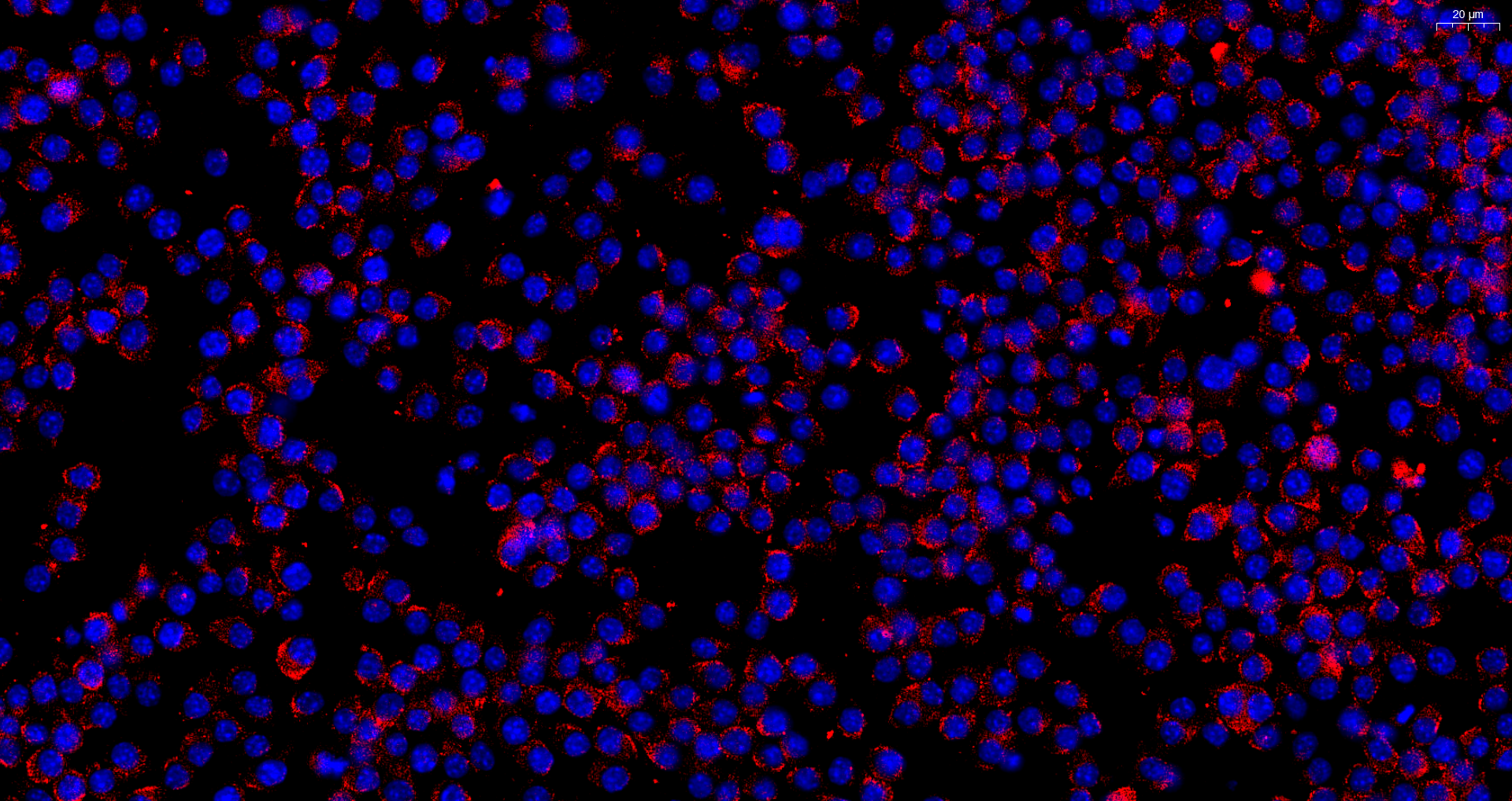

Supplement: Supplementary file 1 [file DataSheet1.ZIP › rawdata(补充材料上传)/figure10G免疫荧光IL6/3.Hesperidin+LPS/RAW IL-6 - Annotation 3_40.0x.tif]

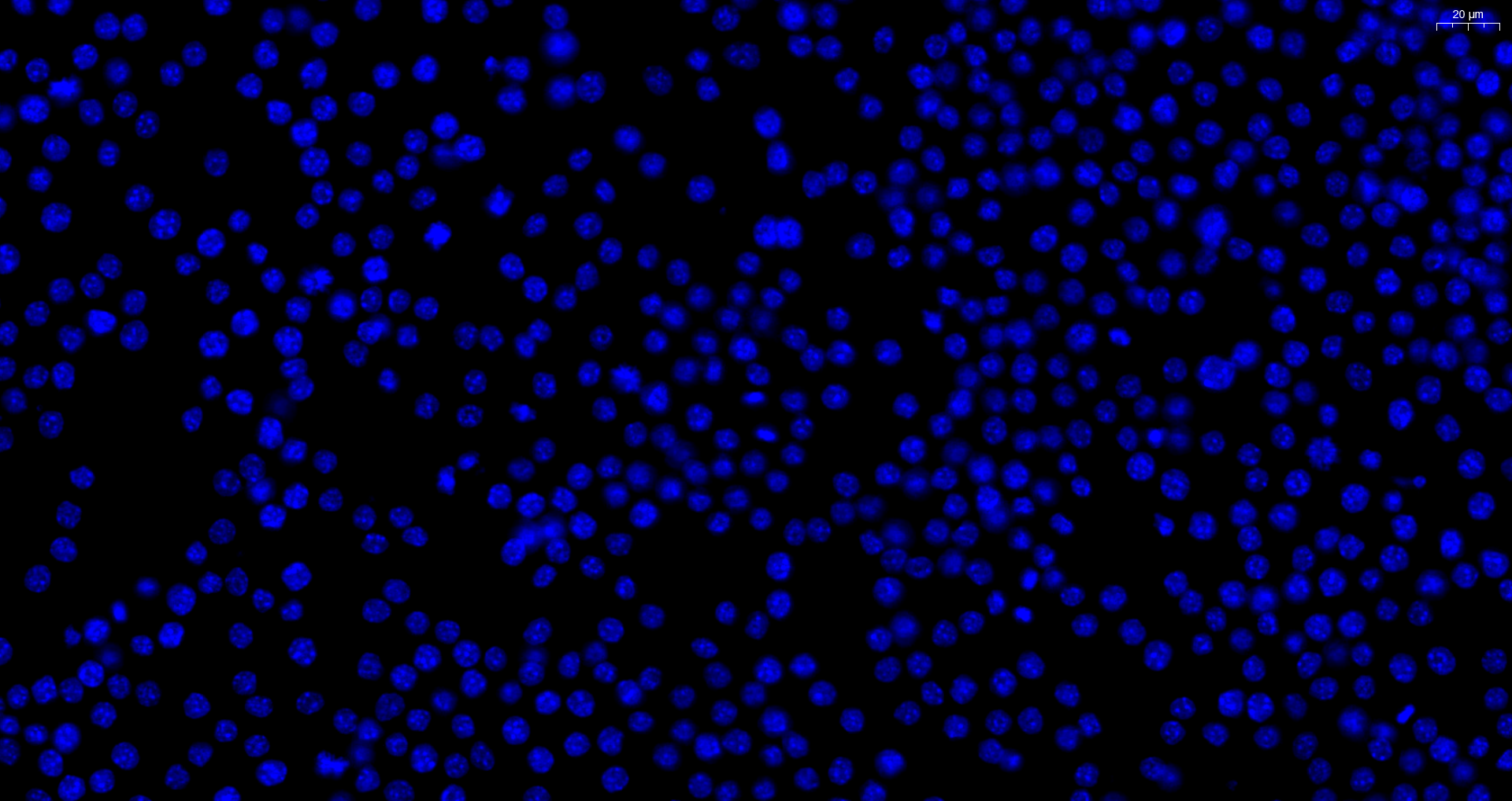

Supplement: Supplementary file 1 [file DataSheet1.ZIP › rawdata(补充材料上传)/figure10G免疫荧光IL6/3.Hesperidin+LPS/DAPI_40.0x.tif]

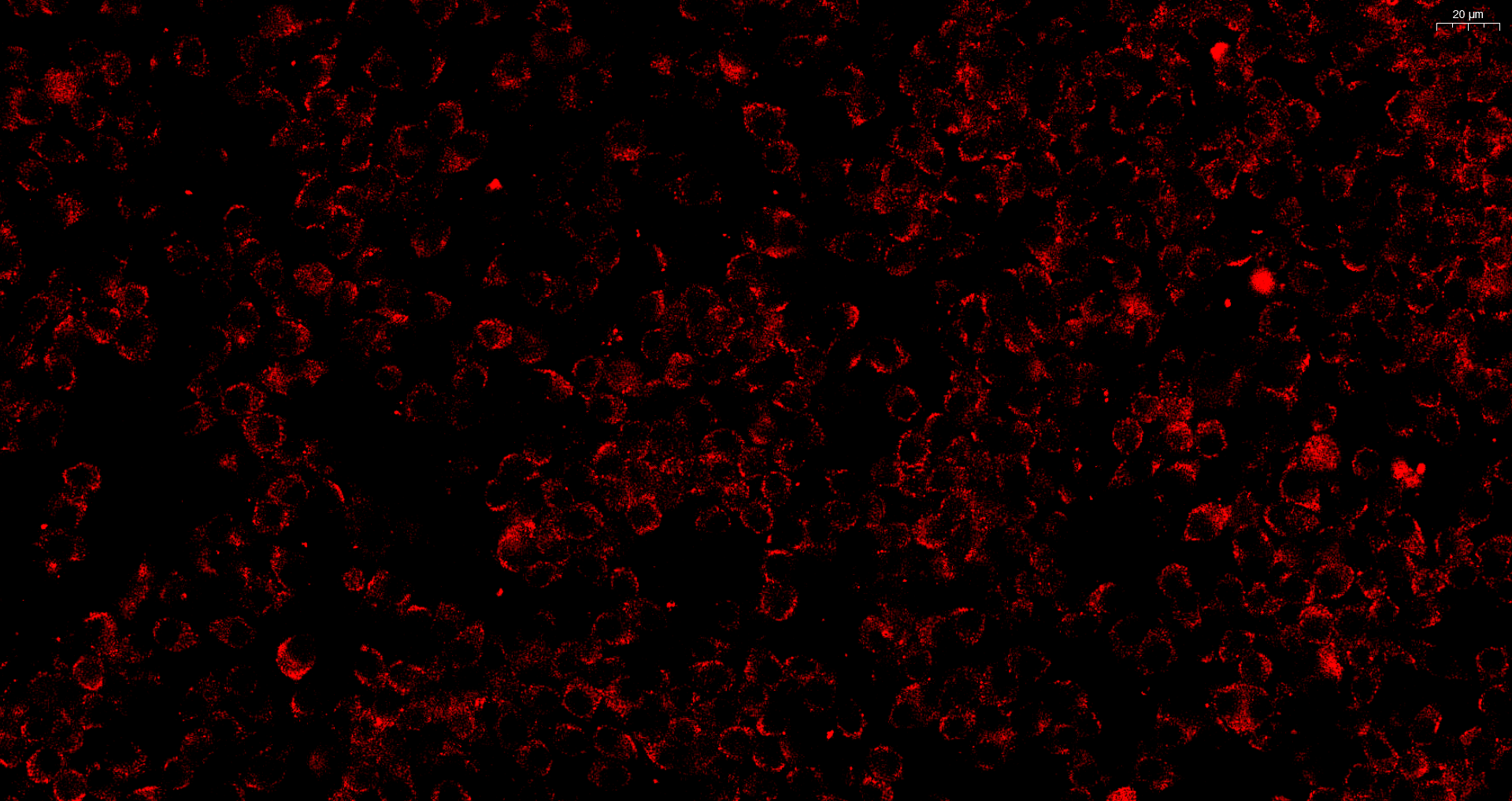

Supplement: Supplementary file 1 [file DataSheet1.ZIP › rawdata(补充材料上传)/figure10G免疫荧光IL6/3.Hesperidin+LPS/IL6_40.0x.tif]

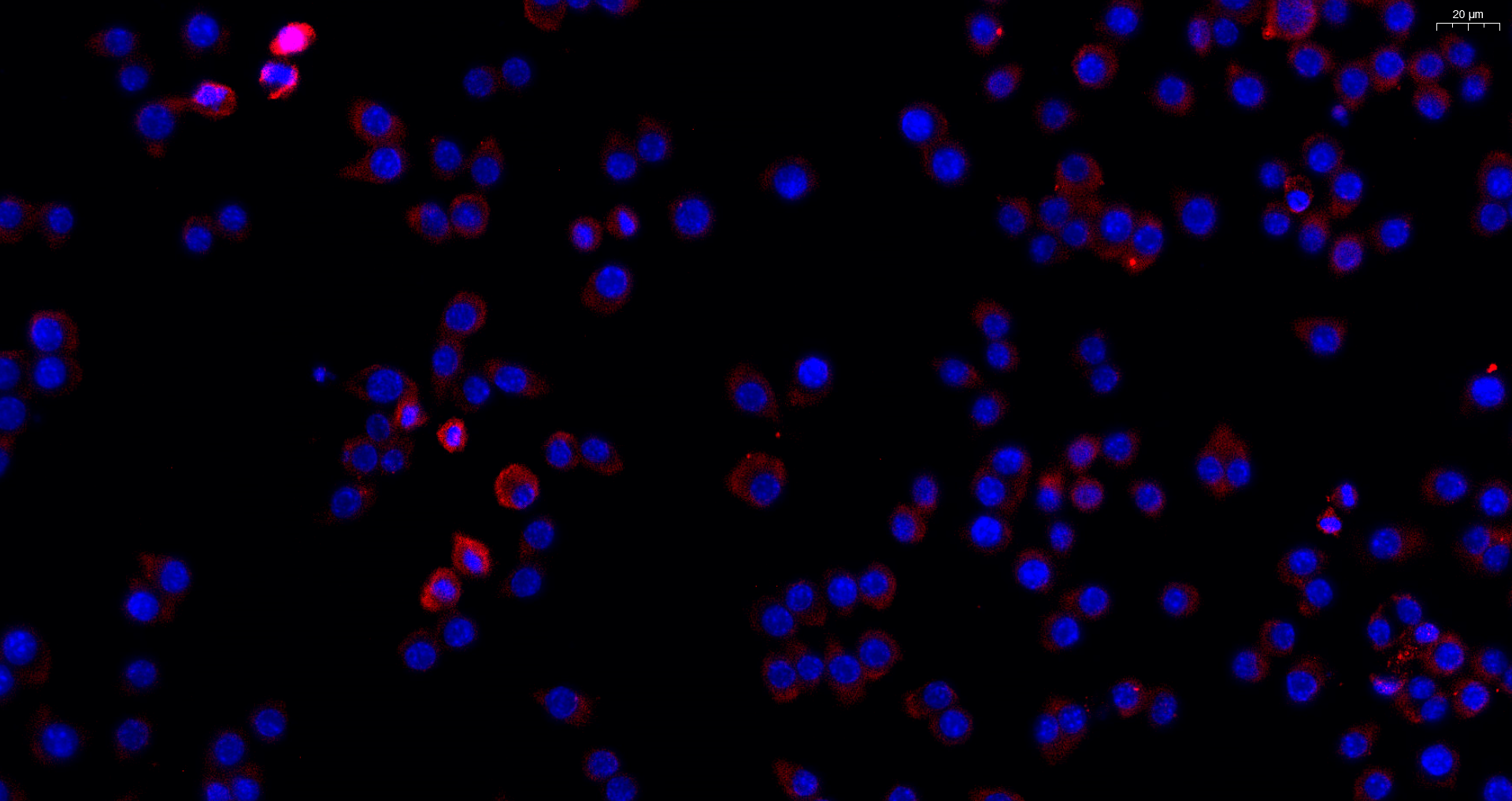

Supplement: Supplementary file 1 [file DataSheet1.ZIP › rawdata(补充材料上传)/figure10H免疫荧光INOS/1.control/RAW inos - Annotation 1_40.0x.tif]

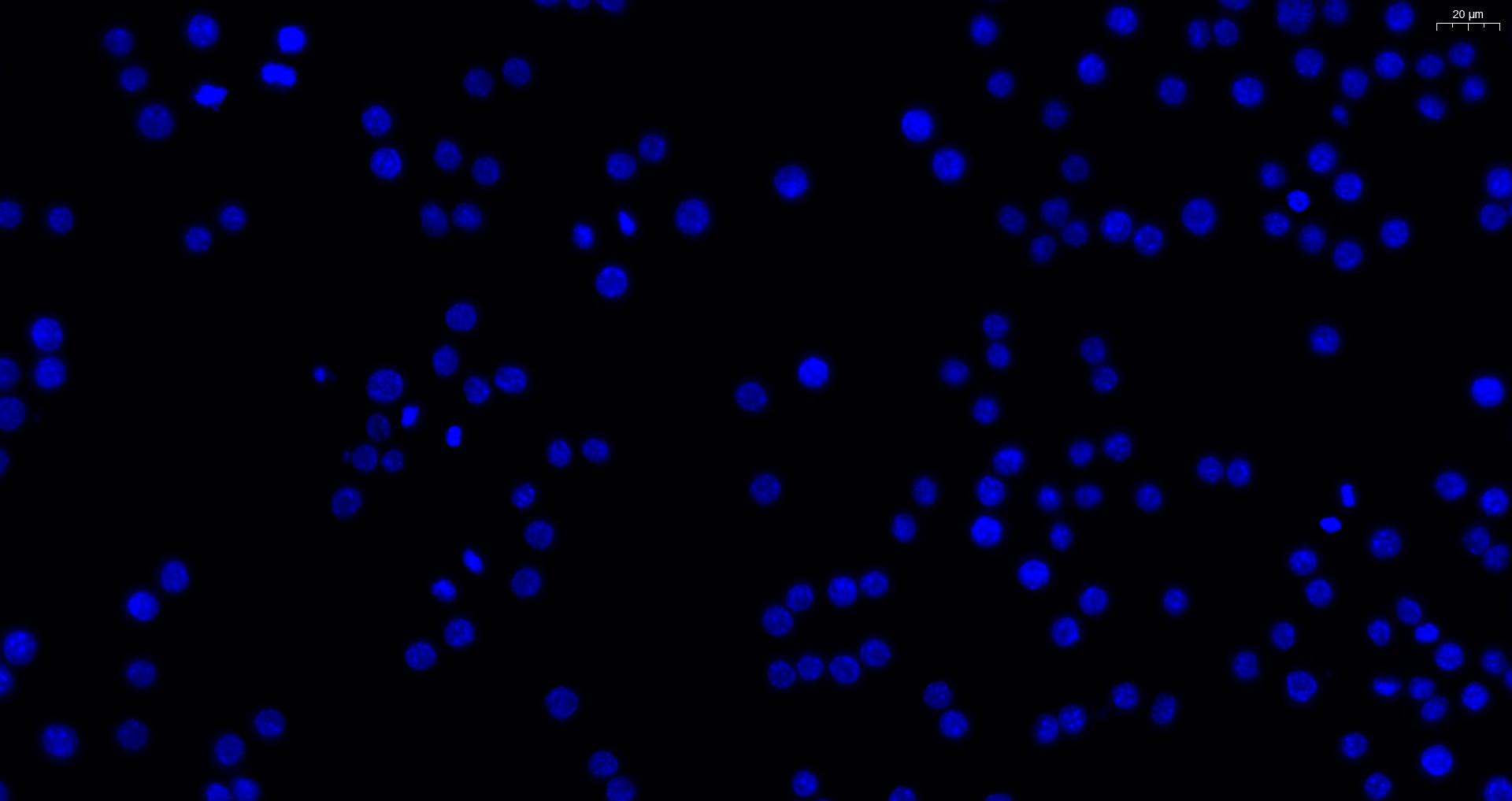

Supplement: Supplementary file 1 [file DataSheet1.ZIP › rawdata(补充材料上传)/figure10H免疫荧光INOS/1.control/DAPI_40.0x.tif]

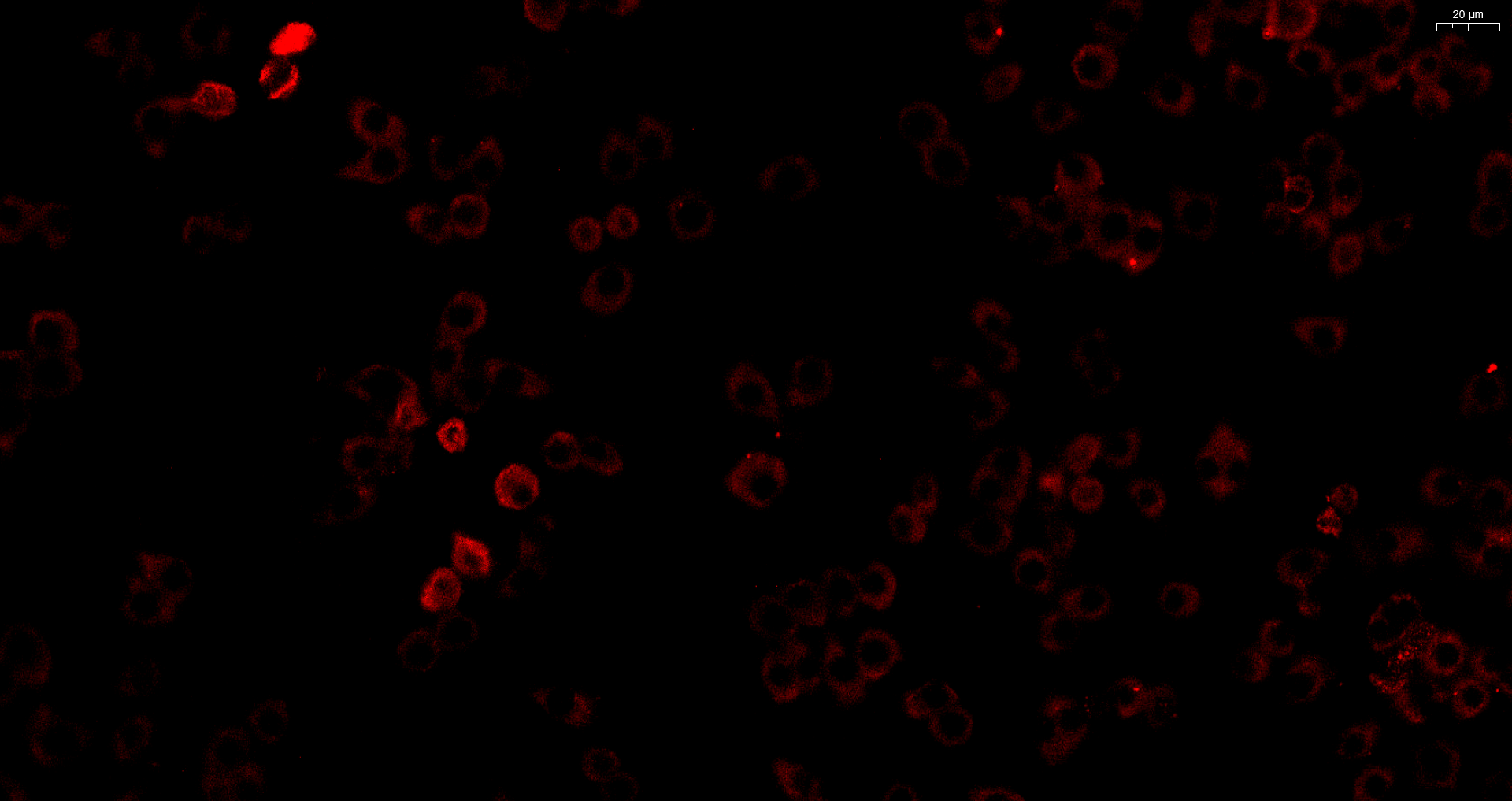

Supplement: Supplementary file 1 [file DataSheet1.ZIP › rawdata(补充材料上传)/figure10H免疫荧光INOS/1.control/INOS_40.0x.tif]

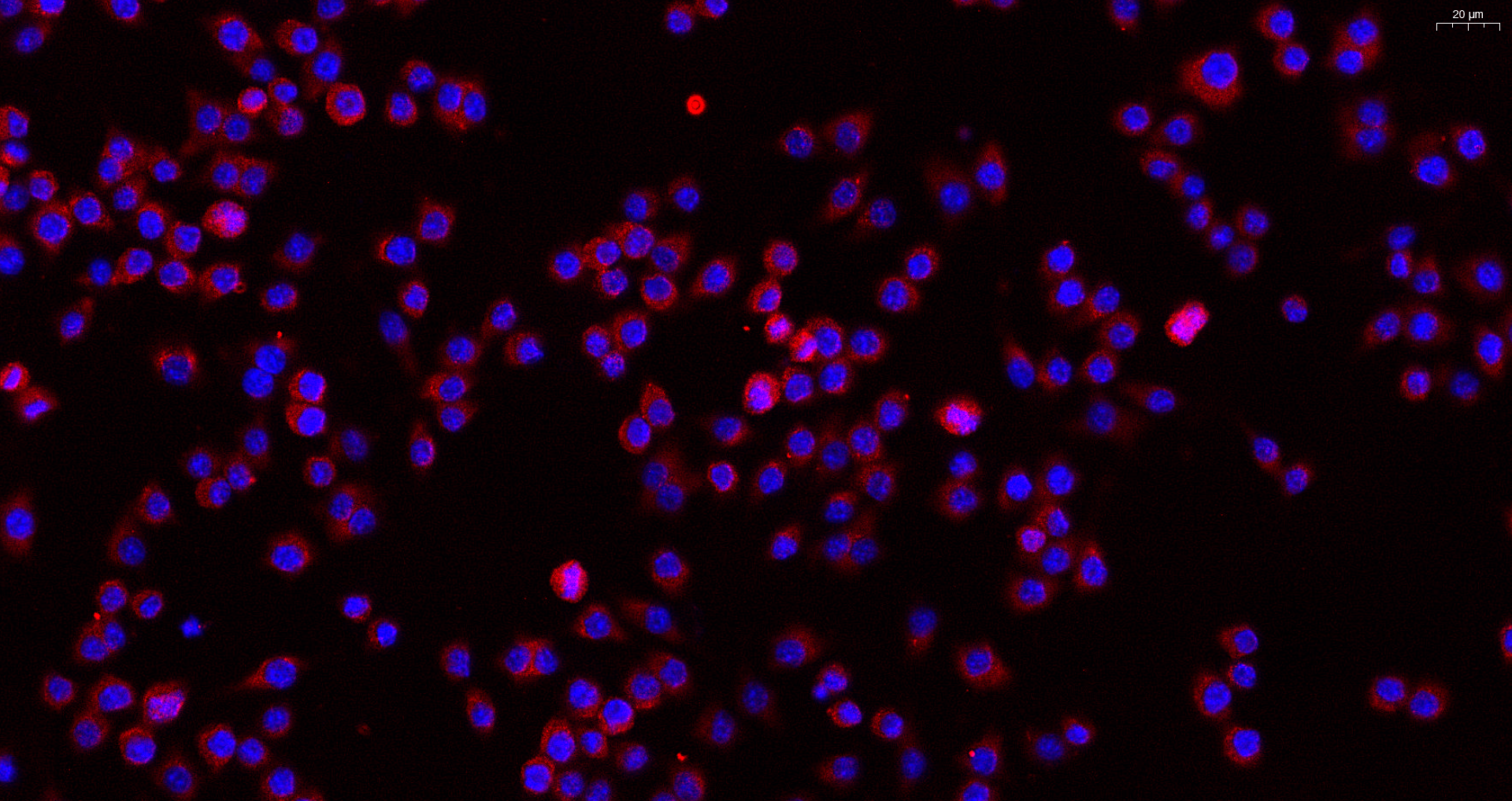

Supplement: Supplementary file 1 [file DataSheet1.ZIP › rawdata(补充材料上传)/figure10H免疫荧光INOS/2.LPS/RAW inos - Annotation 2_40.0x.tif]

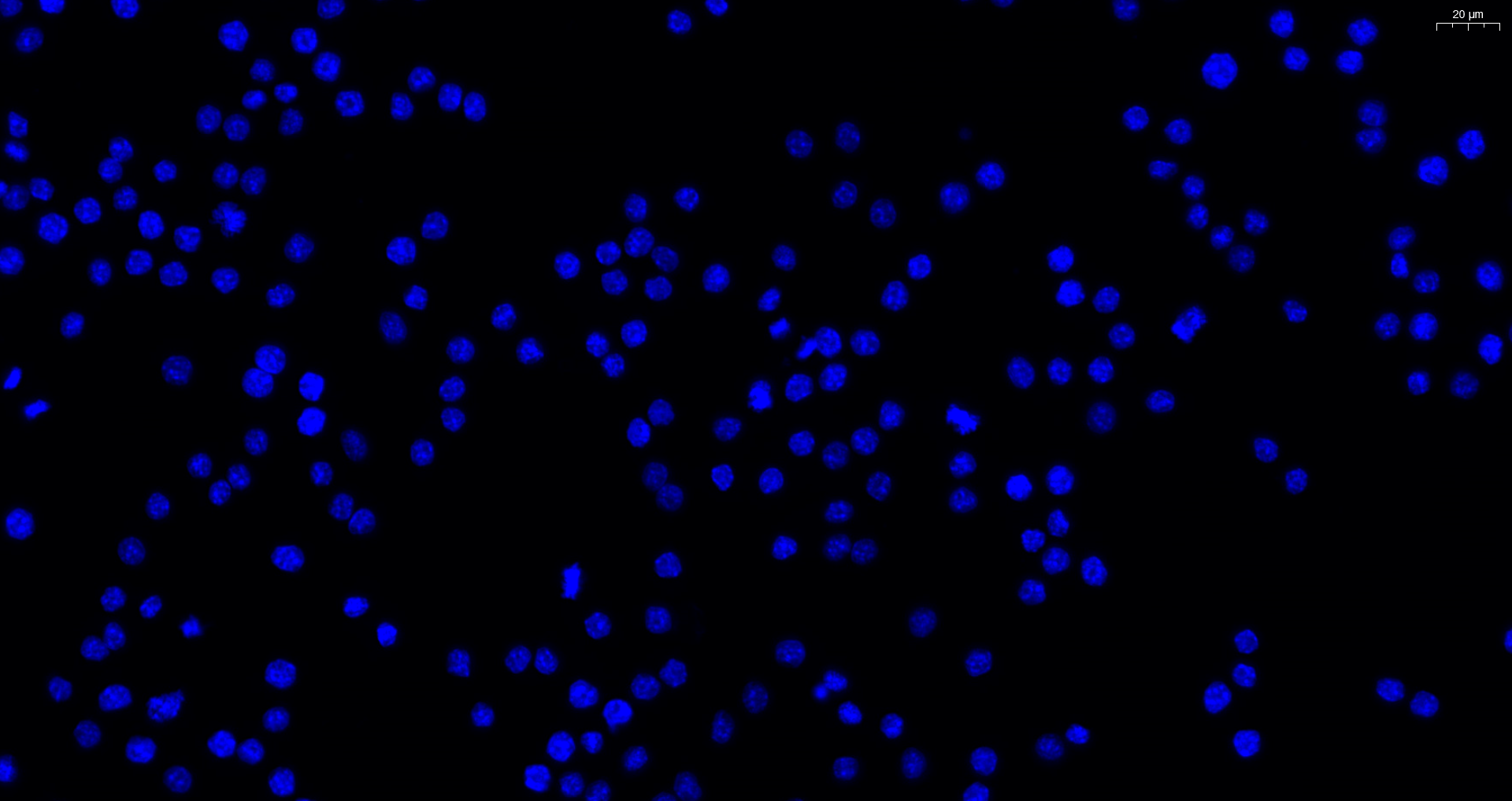

Supplement: Supplementary file 1 [file DataSheet1.ZIP › rawdata(补充材料上传)/figure10H免疫荧光INOS/2.LPS/DAPI_40.0x.tif]

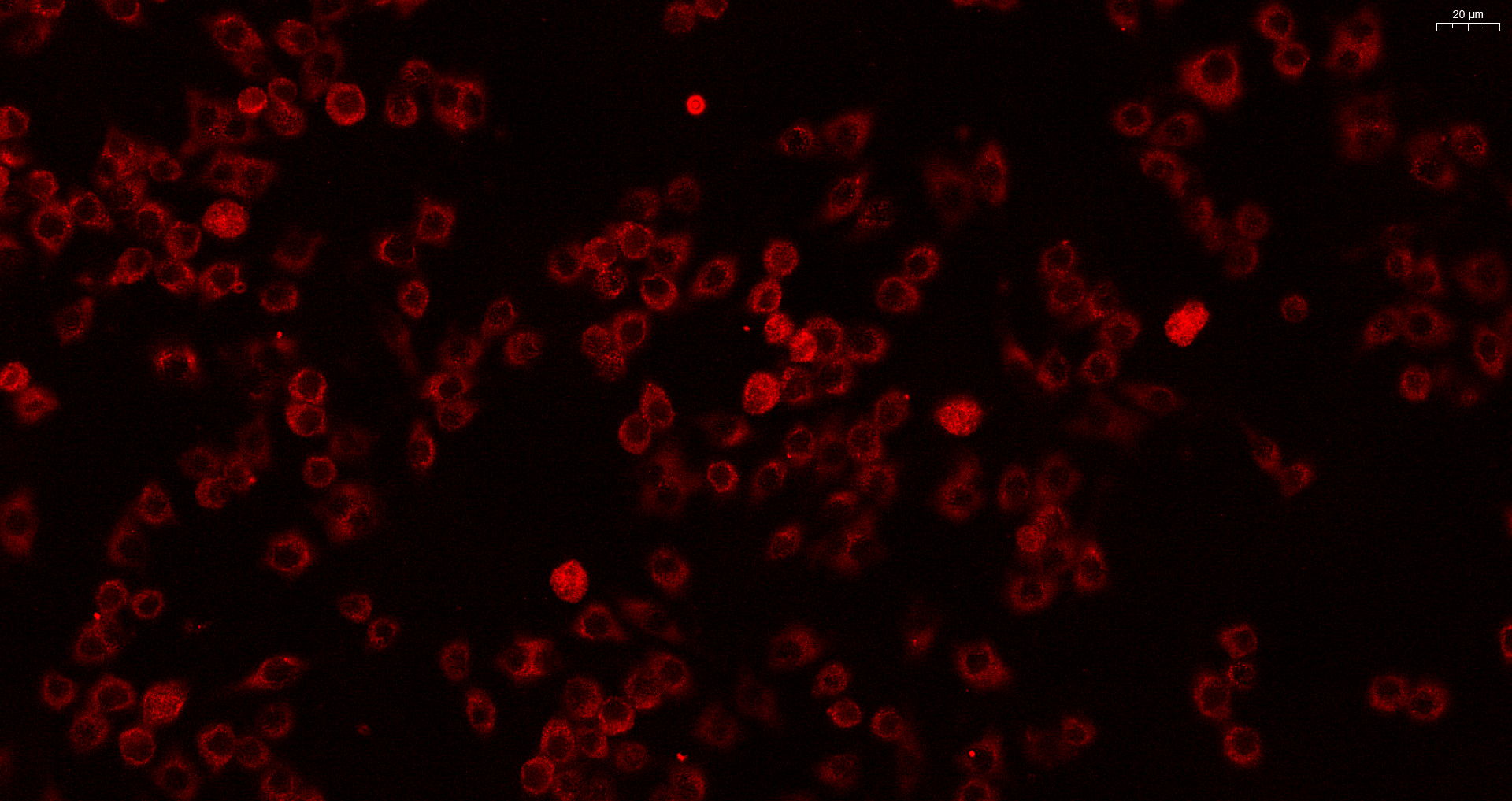

Supplement: Supplementary file 1 [file DataSheet1.ZIP › rawdata(补充材料上传)/figure10H免疫荧光INOS/2.LPS/INOS_40.0x.tif]

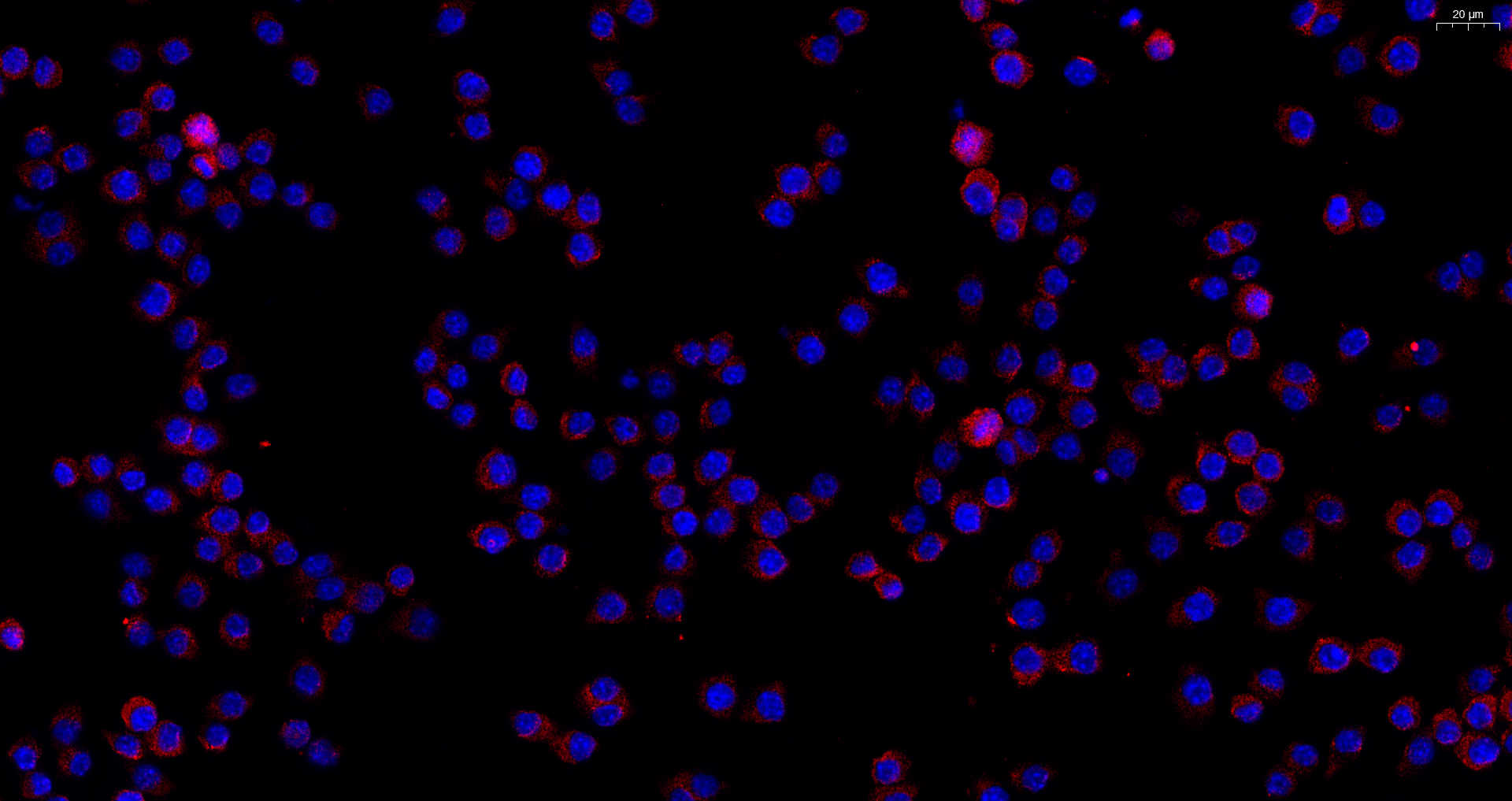

Supplement: Supplementary file 1 [file DataSheet1.ZIP › rawdata(补充材料上传)/figure10H免疫荧光INOS/3.Hesperidin+LPS/RAW inos - Annotation 3_40.0x.tif]

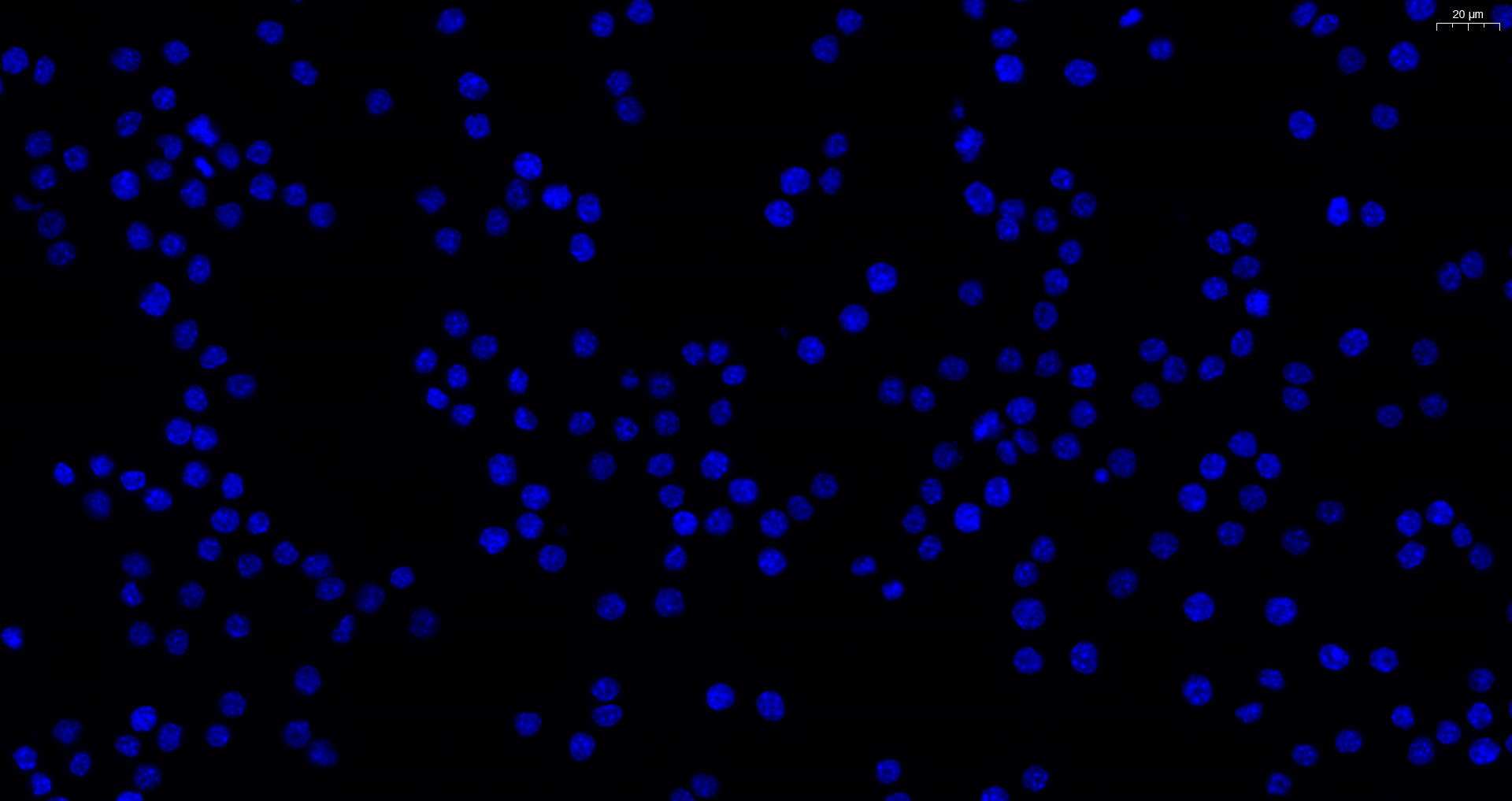

Supplement: Supplementary file 1 [file DataSheet1.ZIP › rawdata(补充材料上传)/figure10H免疫荧光INOS/3.Hesperidin+LPS/DAPI_40.0x.tif]

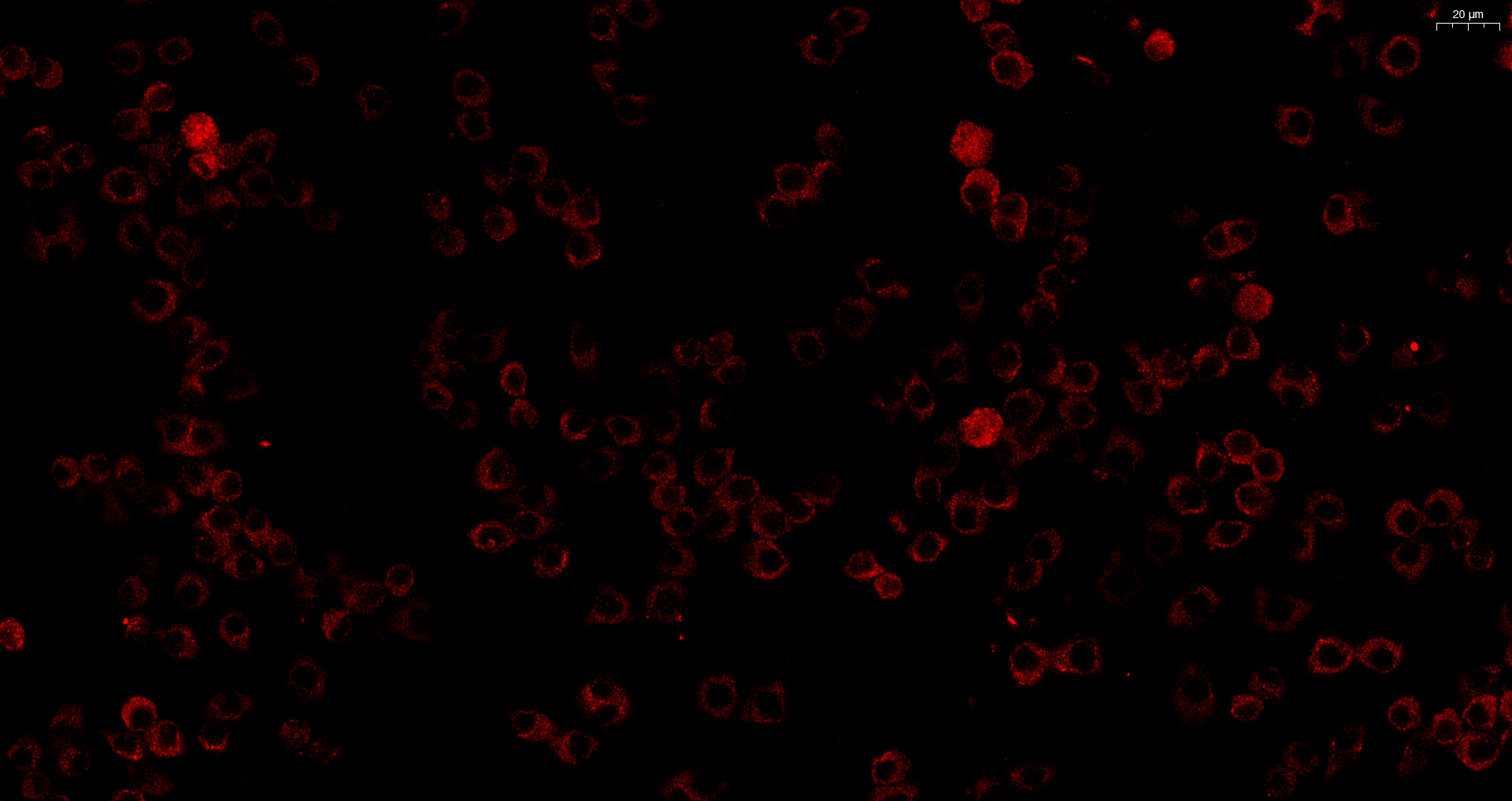

Supplement: Supplementary file 1 [file DataSheet1.ZIP › rawdata(补充材料上传)/figure10H免疫荧光INOS/3.Hesperidin+LPS/INOS_40.0x.tif]
